# Neuronal HLH‐30/TFEB modulates peripheral mitochondrial fragmentation to improve thermoresistance in *Caenorhabditis elegans*

**DOI:** 10.1111/acel.13741

**Published:** 2022-11-23

**Authors:** Shi Quan Wong, Catherine J. Ryan, Dennis M. Bonal, Joslyn Mills, Louis R. Lapierre

**Affiliations:** ^1^ Department of Molecular Biology, Cell Biology and Biochemistry Brown University Providence Rhode Island USA; ^2^ Pathobiology Graduate Program, Division of Biology & Medicine Brown University Providence Rhode Island USA; ^3^ Department of Biology Wheaton College Norton Massachusetts USA; ^4^ Département de Chimie et Biochimie Université de Moncton Moncton New Brunswick Canada; ^5^ New Brunswick Center for Precision Medicine Moncton New Brunswick Canada

**Keywords:** *Caenorhabditis elegans*, HLH‐30/TFEB, mitochondrial dynamics, neuronal signaling, thermoresistance

## Abstract

Transcription factor EB (TFEB) is a conserved master transcriptional activator of autophagy and lysosomal genes that modulates organismal lifespan regulation and stress resistance. As neurons can coordinate organism‐wide processes, we investigated the role of neuronal TFEB in stress resistance and longevity. To this end, the *Caenorhabditis elegans TFEB* ortholog, *hlh‐30*, was rescued panneuronally in *hlh‐30* loss of function mutants. While important in the long lifespan of *daf‐2* animals, neuronal HLH‐30/TFEB was not sufficient to restore normal lifespan in short‐lived *hlh‐30* mutants. However, neuronal HLH‐30/TFEB rescue mediated robust improvements in the heat stress resistance of wildtype but not *daf‐2* animals. Notably, these mechanisms can be uncoupled, as neuronal HLH‐30/TFEB requires DAF‐16/FOXO to regulate longevity but not thermoresistance. Through further transcriptomics profiling and functional analysis, we discovered that neuronal HLH‐30/TFEB modulates neurotransmission through the hitherto uncharacterized protein W06A11.1 by inducing peripheral mitochondrial fragmentation and organismal heat stress resistance in a non‐cell autonomous manner. Taken together, this study uncovers a novel mechanism of heat stress protection mediated by neuronal HLH‐30/TFEB.

## Graphical abstract

Neuronal HLH‐30/TFEB regulates longevity and thermoresistance. Through the uncharacterized protein W06A11.1, it regulates neurotransmission signaling to induce peripheral mitochondrial fragmentation, in turn promoting thermoresistance in a non‐cell autonomous manner.

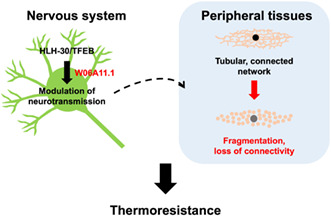

**Abbreviations**
3’UTR: 3’; untranslated region
BP: Biological processes
DCV: Dense core vesicle
DEGs: Differentially expressed genes
ER: Endoplasmic reticulum
UPR^ER^
: Endoplasmic reticulum unfolded protein response
EO: Events observed
FOXO: Forkhead Box O
GO: Gene ontology
GSEA: Gene set enrichment analysis
HSF‐1: Heat shock factor 1
hsp: Heat shock protein
HSR: Heat shock response
IIS: Insulin/insulin‐like growth factor signaling
LogFC: Log_2_ fold change
mTORC1: Mammalian target of rapamycin complex 1
MSigDB: Molecular signature database
NGM: Nematode growth media
NES: Normalized enrichment scores
Non‐Tg: Non‐transgenic
PCA: Principal component analysis
qPCR: Quantitative PCR
ROIs: Regions of interest
Rlog: Regularized log transformed
TFEB: Transcription factor EB
Tg: Transgenic

## 1 INTRODUCTION

Transcription factor EB (TFEB) transcriptionally induces genes of the autophagy and lysosomal pathway, a process that recycles cellular components through lysosomal degradation (Settembre et al., 2011). The transcriptional activity of TFEB is dependent on its nuclear translocation, which is modulated through phosphorylation by mammalian target of rapamycin complex 1 (mTORC1), dephosphorylation by calcineurin, and nuclear export by exportin 1 (Li et al., 2018; Martina et al., 2012; Medina et al., 2015; Napolitano et al., 2018; Roczniak‐Ferguson et al., 2012; Settembre et al., 2012; Silvestrini et al., 2018). In addition to its role as an autophagy and lysosomal inducer, studies in *Caenorhabditis elegans* have uncovered the diverse organismal processes regulated by the TFEB ortholog, HLH‐30, including longevity (Lapierre et al., 2013; Lin et al., 2018), adult reproductive diapause (Gerisch et al., 2020), resistances to heat and oxidative stresses (Lin et al., 2018), starvation (Harvald et al., 2017; O'Rourke & Ruvkun, 2013; Settembre et al., 2013), and pathogenic infection (El‐Houjeiri et al., 2019; Visvikis et al., 2014; Wani et al., 2021). Notably, HLH‐30/TFEB mobilizes different transcriptional programs to regulate these processes, demonstrating its versatile role. How HLH‐30/TFEB orchestrates these systemic responses from and between cell and tissue types remain unresolved and important to ascertain, as such signaling mechanisms are potential intervention targets for modulating aging and stress resistance.

Studies in *C. elegans* have revealed the important and conserved role of the nervous system in integrating stress signals and responses to modulate lifespan extension and organismal stress resistance in a non‐cell autonomous manner (reviewed in Miller et al., 2020). In the regulation of longevity for instance, the rescue of DAF‐16 forkhead Box O (FOXO) transcription factor in neurons partially regulated nonneuronal DAF‐16/FOXO activity and lifespan extension in insulin/insulin‐like growth factor receptor signaling (IIS) defective *daf‐2* mutants (Libina et al., 2003), whereas restoring IIS specifically to neurons abrogated the longevity phenotype of these mutants (Wolkow et al., 2000). In the germlineless *glp‐1* longevity model, lifespan extension mediated by microRNA *mir‐71* in neurons required non‐cell autonomous intestinal DAF‐16/FOXO activity (Boulias & Horvitz, 2012). Neurons are also important for mediating the antagonistic activities of the nutrient sensors TORC1 and AMP‐activated protein kinase on lifespan through the non‐cell autonomous regulation of mitochondrial dynamics by neuropeptides (Zhang et al., 2019).

In other *C. elegans* studies, the coordination of organismal stress responses by neurons have additionally been shown to be coupled to lifespan extension. For instance, constitutive neuronal endoplasmic reticulum (ER) unfolded protein response (UPR^ER^) activation by X‐box‐ binding protein 1 neuronal overexpression improved ER stress resistance and proteostasis, and extended lifespan through peripheral intestinal induction of the UPR^ER^, lysosomal activity, and lipid remodeling (Imanikia, Ozbey, et al., 2019; Imanikia, Sheng, et al., 2019; Taylor & Dillin, 2013). In response to heat stress, neurons release neurotransmitters to other tissues to globally induce the heat shock response (HSR) (Prahlad et al., 2008). Furthermore, neuronal overexpression of the canonical HSR transcription factor heat shock factor 1 (HSF‐1) not only enabled serotonin‐mediated non‐cell autonomous HSR induction in the absence of heat (Tatum et al., 2015) but also additionally conferred resistance to heat stress and lifespan extension (Douglas et al., 2015). When faced with proteotoxic stress, neurons were capable of stimulating proteostasis distally by increasing molecular chaperone production in muscles through a non‐cell autonomous signaling event termed transcellular chaperone signaling (O'Brien et al., 2018). Overall, these findings highlighted that neurons constitute an essential site for lifespan modulation and organismal stress responses through non‐cell autonomous mechanisms, although the exact intercellular and inter‐tissue signaling events are largely undetermined.

Since HLH‐30/TFEB is necessary for most longevity models (Lapierre et al., 2013) and neurons are crucial in regulating longevity and stress resistance (Miller et al., 2020), HLH‐30/TFEB activity in neurons may be important in modulating these organismal phenotypes. Using *C. elegans*, we discovered that neuronal HLH‐30/TFEB regulates both IIS‐dependent longevity and thermoresistance. Neuronal HLH‐30/TFEB mediated thermoresistance via a W06A11.1‐mediated peripheral induction of mitochondria fragmentation through a neurotransmission‐modulatory mechanism, highlighting a beneficial role of mitochondrial fragmentation in heat stress protection.

## 2 RESULTS

### 2.1 Neuronal HLH‐30/TFEB regulates IIS‐dependent longevity

**FIGURE 1:**
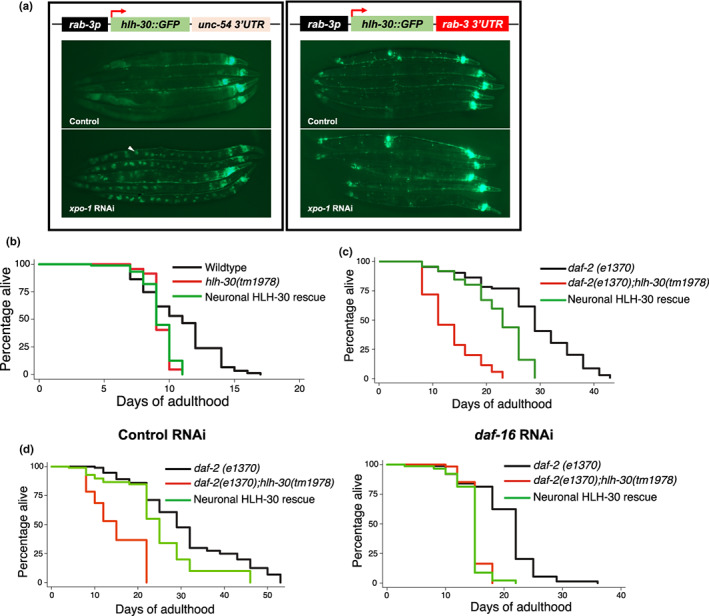
Neuronal HLH‐30/TFEB is a regulator of longevity. (a) (Left panel) Presence and (Right panel) absence of HLH‐30::GFP intestinal misexpression (arrowhead, GFP‐enriched intestinal nucleus) under combinatorial regulation from the *rab‐3* promoter (*rab‐3p*) and *unc‐54* or *rab‐3* 3′ untranslated region (3′UTR), respectively, as detected with RNAi knockdown of the nuclear exportin gene *xpo1*, (b) Lifespan analyses of wildtype, *hlh‐30(tm1978)*, and neuronal HLH‐30/TFEB rescued, *hlh‐30(tm1978)* animals fed OP50 at 25°C. (c, d) Lifespan analyses of *daf2(e1370)*, *daf‐2(e1370);hlh‐30(tm1978)*, and neuronal HLH‐30/TFEB rescued *daf2(e1370);hlh‐30(tm1978)* animals fed (c) OP50 and (d) control RNAi (*L4440*) or RNAi against *daf‐16* at 25°C. Animals were developed on OP50 at 20°C and shifted to 25°C on (a to c) OP50 or (d) bacteria expressing RNAi from day 1 of adulthood. Data are representatives of 3 independent replicates and comparisons were made by Mantel‐Cox log‐rank. See Figures S1 and S2, Tables S1–S3 for additional details on lifespan analyses.

To investigate the activity of HLH‐30/TFEB in neurons, we initially directed panneuronal *hlh‐30::GFP* reporter expression in wildtype *C. elegans* with the *rab‐3* neuronal promoter and the somatic expression‐permitting *unc‐54* 3′ untranslated region (3′UTR) (Kimble & Crittenden, 2007). Corroborating previous reports, we observed misregulation of intestinal transgene expression by this promoter and 3′UTR combination (Gelino et al., 2016; Silva‐García et al., 2019; Wang et al., 2017) as HLH‐30::GFP nuclear enrichment, as induced by knockdown of the nuclear exportin gene *xpo‐1* (Silvestrini et al., 2018), was detected in intestinal cells (Figure 1a). As undesirable nonneuronal expression occludes the ability to examine the neuronal‐specific role of HLH‐30/TFEB, we subsequently drove reporter expression with the *rab‐3* promoter and its corresponding *rab‐3* 3′UTR (Silva‐García et al., 2019 *)* and confirmed neuronal restriction of the transgene by the absence of intestinal nuclear enrichment from *xpo‐*1 knockdown (Figure 1a). We first investigated if neuronal HLH‐30/TFEB plays a role in lifespan regulation. As *hlh‐30(tm1978)* mutants exhibited shortened lifespan in comparison to wildtype animals at 25°C and not 20°C (Lapierre et al., 2013; Lin et al., 2018; Visvikis et al., 2014), we assessed survival at 25°C so that neuronal HLH‐30/TFEB‐dependent benefits on lifespan could be parsed out. In corroboration with previous observations (Lin et al., 2018; Visvikis et al., 2014), *hlh‐30(tm1978)* mutants exhibited reduced lifespan in comparison to wildtype animals at 25°C (Figure 1b, Figure S1a–e and Table S1). Additionally, neuronal HLH‐30/TFEB rescue in these mutants conferred no significant lifespan improvements in several transgenic lines apart from one line whereby lifespan was extended (Figure 1b, Figure S1a–e and Table S1), which was not due to higher neuronal *hlh‐30* expression (Figure S1f). Altogether, these observations suggest that re‐expressing HLH‐30/TFEB in the neurons is not sufficient to restore normal lifespan.

Since systemic HLH‐30/TFEB was previously demonstrated to be essential for lifespan extension in several longevity paradigms (Lapierre et al., 2013; Lin et al., 2018), we next investigated the role of neuronal HLH‐30/TFEB in regulating longevity. As DAF‐16/FOXO was previously reported to co‐regulate IIS‐dependent longevity with HLH‐30/TFEB (Lin et al., 2018), we investigated this in long‐lived *daf‐2(e1370)* IIS mutants in which longevity is mediated by DAF‐16/FOXO activity (Kenyon et al., 1993; Lin et al., 1997). We again performed lifespan analyses at 25°C to maintain consistency so that neuronal HLH‐30/TFEB requirement for normal lifespan and longevity can be compared. *daf2(e1370);hlh‐30(tm1978)* double mutants exhibited reduced lifespan in comparison to long‐lived *daf‐2(e1370)* animals, in line with previous studies of *hlh‐30* loss of function and silencing in *daf‐2* mutants (Figure 1c, Figure S2a, b and Table S2) (Lapierre et al., 2013; Lin et al., 2018). In contrast to observations in *hlh‐30(tm1978)* mutants (Figure 1b), the partial restoration of lifespan by neuronal HLH‐30/TFEB rescue in *daf‐2(e1370);hlh‐30(tm1978)* animals was preserved in several lines (Figure 1c, Figure S2a, b and Table S2), which was abolished with RNAi‐mediated knockdown of *daf‐16* (Figure 1d, Table S3), indicating that neuronal HLH‐30/TFEB mediates longevity in *daf‐2* mutants dependently on DAF‐16/FOXO. Of note, although lifespan can be affected by different bacterial food sources (Stuhr & Curran, 2020), we observed similar lifespan trends from animals fed OP50 or control RNAi in HT115 *Escherichia coli* herein (Figure 1c, d).To determine if differences in *hlh‐30* expression account for disparities in neuronal HLH‐30/TFEB requirements for lifespan extension in *daf‐2(e1370);hlh‐30(tm1978)* and *hlh‐30(tm1978)* mutants, we compared *hlh‐30* expression levels of these animals to their *daf‐2* and wildtype controls. Although *daf‐2* mutants have greater *hlh‐30* expression than wildtype animals (Figure S2c), neuronal HLH‐30/TFEB rescued *daf‐2(e1370);hlh‐30(tm1978)* mutants exhibited similar *hlh‐30* expression to their *daf‐2* controls (Figure S2c), whereas neuronal HLH‐30/TFEB rescued *hlh‐30(tm1978)* mutants had similar or higher *hlh‐30* expressions than their wildtype controls (Figure S1f). This indicated that the robust lifespan extension by neuronal HLH‐30/TFEB in *daf‐2(e1370);hlh‐30(tm1978)* mutants was not due to greater neuronal HLH‐30/TFEB expression. Taken together, these results demonstrated that neuronal HLH‐30/TFEB is not necessarily required for the regulation of normal lifespan but is essential for IIS‐dependent longevity.

### 2.2 Neuronal HLH‐30/TFEB mediates heat stress resistance independently of DAF16/FOXO

**FIGURE 2:**
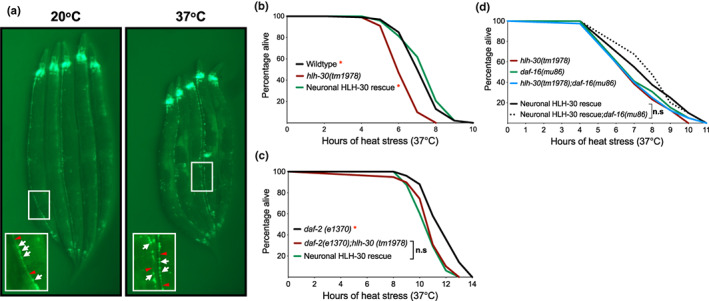
Neuronal HLH‐30/TFEB mediates resistance to heat stress. (a) HLH‐30::GFP in neurons at control conditions (20°C) and following 3 h of heat stress at 37°C. Regions of GFP‐marked ventral nerve cords are shown in enlarged insets with neuronal cell bodies (white arrows) and axonal tracts (red arrowheads) as indicated. (b, c) Survival analyses of neuronal HLH‐30/TFEB rescued animals in comparison with (b) wildtype, *hlh‐30(tm1978)*, (c) *daf‐2(e1370)*, and *daf‐2(e1370);hlh‐30(tm1978)* controls at 37°C heat stress. (d) Survival analyses of neuronal HLH‐30/TFEB rescued *hlh‐30(tm1978)* animals in in the absence (black) and presence (black dotted) of *daf16(mu86)* loss of function at 37°C heat stress. Animals were developed at 20°C and shifted to 37°C to induce heat stress at day 1 of adulthood. Data are representatives of 2–4 independent replicates and comparisons were made by Mantel‐Cox log‐rank (*n* = 78–200/strain; n.s, *p* ≥ 0.05; *, *p* < 0.05; in comparison to (b) *hlh‐30(tm1978)*, (c) *daf2(e1370);hlh‐30(tm1978)* and (d) neuronal HLH‐30/TFEB rescued animals). See Figure S3 for heat stress survival analyses on additional neuronal HLH‐30/TFEB rescued lines.

The relationship between thermoresistance and lifespan extension was demonstrated by improved thermotolerance from pro‐longevity mutations and lifespan‐extending effects of transient heat stress exposure in *C. elegans* (Butov et al., 2001; Kumsta et al., 2017; Lithgow et al., 1995; Michalski et al., 2001). As HLH‐30/TFEB is systemically required for thermotolerance (Lin et al., 2018) and long‐lived *daf‐2(e1370)* mutants are more thermotolerant than wildtype animals (Lithgow et al., 1995), we wanted to investigate if neuronal HLH‐30/TFEB mediates thermoresistance since it regulates IIS‐dependent longevity. Corroborating previous observations (Lin et al., 2018), animals expressing ubiquitous or neuronal HLH‐30::GFP exhibited HLH‐30/TFEB nuclear enrichment following exposure to acute heat stress at 37°C (Figure 2a, Figure S3a, b). Notably, *hlh‐30(tm1978)* mutants exhibited compromised survival in comparison to wildtype animals with prolonged heat stress, which was mitigated by the neuronal rescue of HLH‐30/TFEB (Figure 2b, Figure S3c, d). Additionally, overexpression of neuronal HLH‐30/TFEB enhanced the survival of wildtype animals to heat stress (Figure S3g). Surprisingly, we found that neuronal HLH‐30/TFEB rescue failed to improve the reduced survival of *daf‐2(e1370);hlh‐30(tm1978)* double mutants to heat stress (Figure 2c, Figure S3e, f). In sum, our observations demonstrate that neuronal HLH‐30/TFEB is important for heat stress resistance in wildtype but not in long‐lived *daf‐2(e1370)* animals.

Interestingly, our findings indicate that there are differential requirements for neuronal HLH‐30/TFEB in regulating longevity and thermoresistance in wildtype and long‐lived genetic backgrounds. Moreover, the lack of survival improvements with neuronal HLH‐30/TFEB rescue in *daf‐2(e1370);hlh‐30(tm1978)* double mutants despite DAF‐16/FOXO‐dependent lifespan extension (Figure 1d, Table S3) suggests that neuronal HLH‐30/TFEB does not regulate thermoresistance through DAF‐16/FOXO, in line with previous findings that these transcription factors mediate heat stress protection through separate pathways (Lin et al., 2018). Indeed, loss of function *daf‐16(mu86)* did not dampen the survival of neuronal HLH‐30/TFEB rescued animals to heat stress in non*daf‐2(e1370)* animals (Figure 2d). Taken together, these findings demonstrated that neuronal HLH‐30/TFEB requires DAF‐16/FOXO to regulate longevity but not thermoresistance.

### 2.3 Neuronal HLH‐30/TFEB mediates thermoresistance via W06A11.1

**FIGURE 3:**
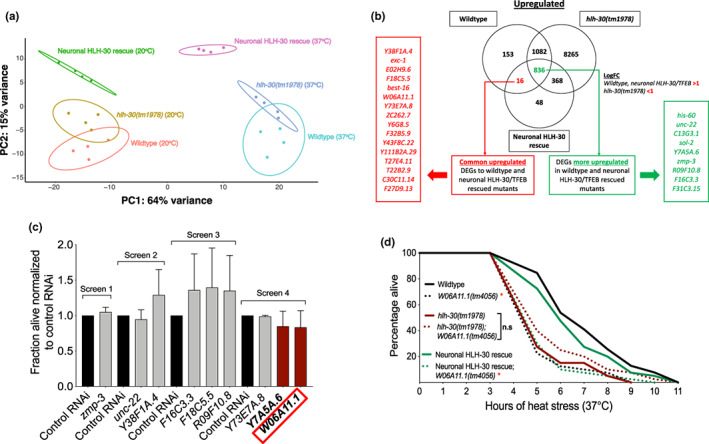
Neuronal HLH‐30 mediates thermoresistance through *W06A11.1*. (a) Principal component analysis (PCA) plot of wildtype, *hlh‐30(tm1978)*, and neuronal HLH‐30/TFEB rescued animals based on the regularized log transformed gene count tables from the RNAseq analysis obtained from 20°C (control conditions) and 37°C (heat stress). (b) Genes upregulated by 37°C (heat stress) in comparison to 20°C (control conditions) for each genotype were overlapped to extract significant heat stress‐induced differentially expressed genes (adjusted *p* < 0.05) unique to or more upregulated (Log_2_ fold change [LogFC] thresholds applied as indicated) in wildtype and neuronal HLH‐30/TFEB rescued animals than *hlh‐30(tm1978)* mutants. (a and b) Animals from 4 independent replicates were developed at 20°C to day 1 of adulthood and harvested for RNA after 3 h of further growth at 20°C (control conditions) or 37°C (heat stress). (c) Neuronal HLH‐30/TFEB rescued animals were fed RNAi bacteria to knockdown heat stress‐upregulated genes for 48 h following development at 20°C to day 1 of adulthood, and scored for survival after 7 h of 37°C heat stress (*n* = 88–93/RNAi; fraction alive normalized to control RNAi per screen). Data are representative of 2 independent replicates. (d) Survival analyses of wildtype, *hlh‐30(tm1978)*, and neuronal HLH‐30/TFEB rescued animals in the absence (solid lines) and presence (dotted lines) of *W06A11.1(tm4056)* loss of function at 37°C heat stress. Animals were developed at 20°C to day 1 of adulthood and exposed to 37°C heat stress until death. Data are representative of 2 independent replicates and comparisons were made by Mantel‐Cox log‐rank (*n* = 79–80/strain; n.s, *p* ≥ 0.05; *, *p* < 0.05; *W06A11.1(tm4056)* compared to control per genotype).

To gain insights into mechanisms mobilized by neuronal HLH‐30/TFEB to mediate heat stress resistance, we compared systemic RNAseq transcriptomics profiles of wildtype, *hlh‐30(tm1978)*, and neuronal HLH‐30/TFEB rescued animals from heat stressed to control conditions. Across all genotypes, heat stress induced transcriptional changes including the expected upregulation of the heat shock protein (*hsp)* genes (Lee et al., 1996; Morimoto, 1998) (Figure 3a, Figure S4a, b). We performed gene set enrichment analysis (GSEA) of these transcriptional changes for each genotype to identify gene ontology (GO) biological processes (BP) terms enriched in wildtype and neuronal HLH‐30/TFEB animals but not in *hlh‐30(tm1978)* mutants to identify systemic heat responsive processes dependent on neuronal HLH‐30/TFEB. Although the most significantly enriched terms of “translational initiation,” “positive regulation of cell migration by vascular endothelial growth factor signaling pathway,” and “positive regulation of endothelial cell chemotaxis by VEGF‐activated vascular endothelial growth factor receptor signaling pathway” were commonly upregulated by heat stress across the genotypes, we did not uncover any processes enriched only in wildtype and neuronal HLH‐30/TFEB animals (Figure S4c). Interestingly, GO BP terms relating to neuronal processes such as “protein localization to presynapse” and “axo dendritic protein transport” were most significantly enriched with heat stress in neuronal HLH‐30/TFEB animals, suggesting a cell‐autonomous role of neuronal HLH‐30/TFEB in modulating neuronal function in response to heat stress (Figure S4c). Notably, we did not find significant enrichments of any autophagy‐related GO BP terms with heat stress in wildtype and neuronal HLH‐30/TFEB rescued animals despite HLH‐30/TFEB's role as the master transcriptional activator of autophagy (Figure S5a), suggesting that autophagy may not be transcriptionally induced, at least not at the duration of heat stress investigated herein.

As we were unable to delineate shared global changes between wildtype and neuronal HLH‐30/TFEB animals, we sought to identify the specific genes which exhibited common heat stress‐induced differential expression in both genotypes in comparison to *hlh‐30(tm1978)* mutants. Heat stress‐induced up‐ and downregulated differentially expressed genes (DEGs) identified per genotype were subsequently overlapped between genotypes to extract DEGs unique to or which exhibited a greater expression change in wildtype and neuronal HLH‐30/TFEB rescued animals relative to *hlh‐30(tm1978)* mutants (Figure 3b, Figure S5b). For these analyses, we applied thresholds for adjusted *p*‐values of significance and Log_2_ fold changes and derived a total of 25 upregulated and 9 downregulated DEGs (Figure 3b, Figure S5b). We were unable to perform GSEA or GO enrichment analyses of these DEGs due to the small number and the lack of functional annotation for many of them.

To uncover new neuronal HLH‐30/TFEB‐dependent modulators of heat stress resistance, we first performed gene knockdowns of upregulated DEGs with available RNAi clones on neuronal HLH‐30/TFEB rescued animals to identify promising functional targets for subsequent comprehensive assessment (Figure 3c). As *hlh‐30(tm1978)* mutants exhibited disparate survival from wildtype and neuronal HLH‐30/TFEB rescued animals at the 7th hour of heat stress (Figure 2b, d, Figure S3c, d), we reasoned that detrimental effects of gene knockdowns on survival would be distinguishable at this timepoint from our initial screens. Although we did not observe significant reductions of survival to heat stress at the timepoint examined, genes *Y7A5A.6* and *W06A11.1* exhibited trends in dampened survival with knockdown. We followed up on a comprehensive analysis of W06A11.1 since its expression was originally reported in the muscle (Fox et al., 2007), a tissue that can modulate lifespan and proteostasis in response to neuronal signaling (Burkewitz et al., 2015; Garcia et al., 2007; O'Brien et al., 2018; Prahlad & Morimoto, 2011; Silva et al., 2013; Tatum et al., 2015; van Oosten‐Hawle et al., 2013; Zhang et al., 2019). Of note, our findings of *hlh‐30*‐dependent upregulation of *W06A11.1* with heat stress corroborated previous transcriptomics comparisons between heat‐stressed wildtype and *hlh‐30(tm1978)* mutants (Lin et al., 2018). We found that both loss of function *W06A11.1(tm4056)* and RNAi‐mediated knockdown of *W06A11.1* compromised the survival of wildtype and neuronal HLH‐30/TFEB rescued animals but not of *hlh‐30(tm1978)* mutants during heat stress (Figure 3d, Figure S7a). Interestingly, the lack of these robust depletions in survival with *W06A11.1* knockdown in our initial screens (Figure 3c) suggests that other screened DEGs may also be functional modulators of thermoresistance which did not pass the threshold of detection at the timepoint examined herein. As such, these genes remain possible targets for future studies to explore. Taken together, our findings indicated that neuronal HLH‐30/TFEB mediates thermoresistance dependently of the novel modulator, W06A11.1.

### 2.4 Neuronal HLH‐30/TFEB induces peripheral mitochondrial fragmentation

**FIGURE 4:**
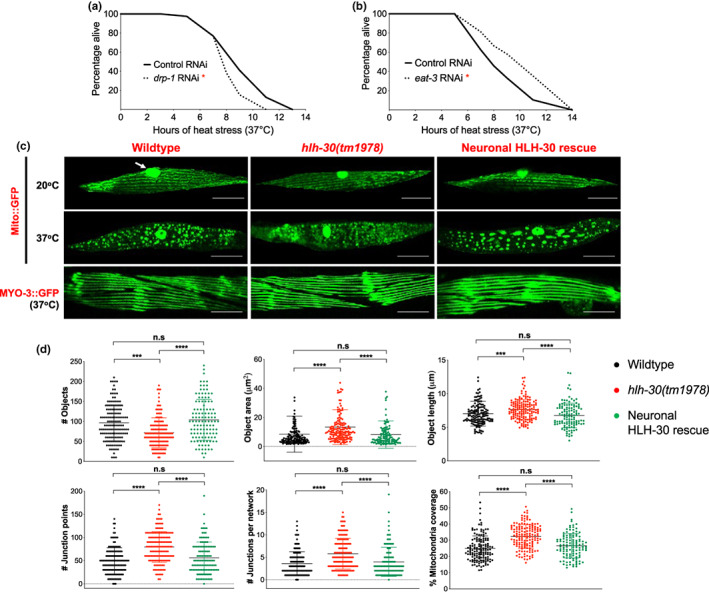
Neuronal HLH‐30/TFEB mediates thermoresistance through peripheral mitochondrial fragmentation. (a and b) Survival analyses of wildtype animals fed control RNAi (*L4440*, solid lines) or RNAi against (a) *drp‐1* and (b) *eat‐3* (dotted lines) at 37°C heat stress. Animals developed at 20°C to the L4 larval stage on OP50 were transferred onto bacteria expressing RNAi for 48 h and exposed to 37°C heat stress until death. Data are representatives of 2 independent replicates (*n* = 89–180/RNAi) and comparisons were made by Mantel‐Cox log‐rank (*, *p* < 0.05; in comparison to control RNAi). (c) Representative images of muscle mitochondrial morphology with the body wall muscle mitochondrial reporter (Mito::GFP) and of muscle morphology with the body wall muscle myosin reporter (MYO‐3::GFP) in wildtype, *hlh‐30(tm1978)*, and neuronal HLH‐30/TFEB rescued animals at 20°C (arrow, GFP in muscle nuclei) or after 37°C heat stress for 3 h. Scale bars = 20 μM. (d) Analysis of mitochondrial connectivity in wildtype, *hlh‐30(tm1978)*, and neuronal HLH‐30/TFEB rescued animals after 37°C heat stress for 3 h. (c, d) Animals were developed at 20°C to day 1 of adulthood and exposed to 37°C heat stress for 3 h. Data are representatives of 4 independent replicates (per strain; *n* = 40, number of ROIs = 117–150) and comparisons were made by Kruskal–Wallis for each mitochondrial feature (presented as mean ± SD; n.s, *p* ≥ 0.05; ***, *p* < 0.001; ****, *p* < 0.0001).

Heat stress was previously found to cause increased mitochondrial fragmentation in muscles (Chen et al., 2021; Machiela et al., 2020; Momma et al., 2017), and disrupting mitochondrial fission and fusion were detrimental and beneficial to heat stress resistance, respectively, suggesting that mitochondrial fragmentation is mechanistically important for thermoresistance (Machiela et al., 2020). Given the reported muscle expression of *W06A11.1* (Fox et al., 2007), and that neuronal signaling can induce distal mitochondrial fragmentation in muscles (Burkewitz et al., 2015; Zhang et al., 2019), we wondered if neuronal HLH‐30/TFEB mediates thermoresistance through muscle mitochondrial fragmentation and if this is W06A11.1‐dependent. To investigate this, we first confirmed the functional importance of mitochondrial fragmentation in heat stress resistance as knocking down the mitochondrial fission and fusion genes, *drp‐1* and *eat‐3*, respectively, dampened and improved the survival of wildtype animals to heat stress (Figure 4a, b). Using a body wall muscle‐targeted, GFP‐tagged mitochondrial reporter (Mito::GFP) (Sarasija & Norman, 2015), we further compared mitochondrial morphologies on either gene knockdown by quantifying various mitochondrial morphological parameters using MitoMAPR (Zhang et al., 2019). Corroborating the functional observations on heat stress survival (Figure 4a, b), mitochondrial fragmentation exhibited overall reduction and increase with *drp‐1* and *eat‐3* knockdown, respectively, during heat stress although we do not observe any morphological differences at control conditions (Figure S6a, b).

To determine if mitochondrial fragmentation is equally important for neuronal HLH‐30/TFEB‐dependent heat stress resistance, we further disrupted either gene in neuronal HLH‐30/TFEB rescued animals. Although *drp‐1* or *eat‐3* knockdown had unexpectedly no effect on the thermoresistance of these animals (Figure S6c), knockdowns of other mitochondrial fission genes (*fis‐1*, *fis‐2*, *mff‐1*, *mff‐2*) uniformly compromised their survival to heat stress (Figure S6d), indicating that mitochondrial fragmentation is functionally required by neuronal HLH‐30/TFEB for mediating heat stress protection. To confirm this, we again utilized the Mito::GFP reporter to compare mitochondrial morphology between wildtype, *hlh‐30(tm1978)*, and neuronal HLH‐30/TFEB rescued animals. Corroborating previous observations (Chen et al., 2021; Machiela et al., 2020; Momma et al., 2017), muscle mitochondria were reticular at 20°C but exhibited extensive fragmentation following heat stress (Figure 4c). As mitochondrial fragmentation often preludes cell death (Suen et al., 2008), we investigated if heat stress additionally induced muscle degeneration. Using a MYO‐3::GFP‐expressing strain (Campagnola et al., 2002), we observed that this was not the case as the continual striated morphology of myosin heavy chain filaments was not compromised by heat stress (Figure 4c). In quantifying the extents of heat stress‐induced mitochondrial fragmentation between the genotypes using MitoMAPR (Zhang et al., 2019), we found that heat stressed *hlh‐30(tm1978)* mitochondria had lower counts but higher areas and length, suggesting reduced breakdown of mitochondrial tubularity (Figure 4d). Furthermore, increased junction points, junctions per mitochondrial network, and mitochondrial coverages, suggest higher mitochondrial network connectivity in these mutants (Figure 4d). Altogether, this indicates that heat stress‐induced mitochondrial fragmentation in the muscles of *hlh‐30(tm1978)* mutants were reduced in comparison to wildtype and neuronal HLH‐30/TFEB rescued animals. Taken together, these findings demonstrate that neuronal HLH‐30/TFEB mediates thermoresistance by non‐cell autonomously inducing mitochondrial fragmentation.

### 2.5 W06A11.1 induces mitochondrial fragmentation to mediate heat stress resistance

**FIGURE 5:**
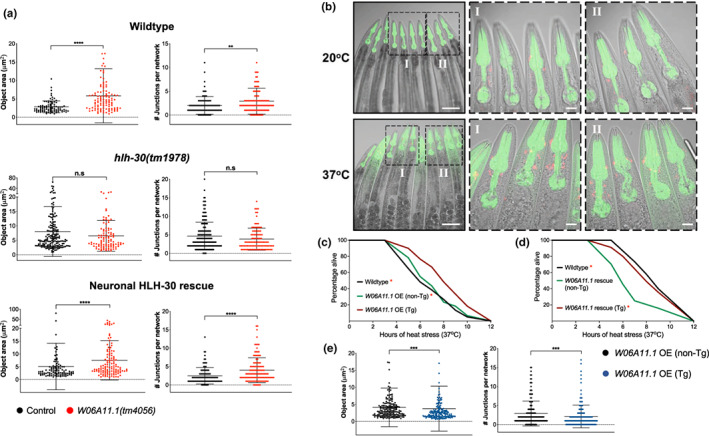
*W06A11.1* induces non‐cell autonomous mitochondrial fragmentation to mediate thermoresistance. (a) Analysis of mitochondrial connectivity in the presence and absence of *W06A11.1(tm4056)* loss of function after 37°C heat stress for 3 h in wildtype, *hlh‐30(tm1978)*, and neuronal HLH‐30/TFEB rescued animals (see Figures S7b–d for additional mitochondrial quantification). Data are representatives of 3 independent replicates (per strain; *n =* 30, number of ROIs = 88–171) and comparisons were made by Mann–Whitney for each mitochondrial feature (presented as mean ± SD; n.s, *p* ≥ 0.05; *, *p* < 0.05; **, *p* < 0.01; ***, *p* < 0.001; ****, *p* < 0.0001). (b) Transgenic (Tg) wildtype animals overexpressing extrachromosomal W06A11.1::DsRed (*W06A11.1* OE) at 20°C (control conditions) and after 37°C heat stress for 5 h. Scale bars = 100 μM, images in left column; scale bars = 20 μM, enlarged insets of head regions (I and II). Survival analyses of wildtype, with non‐//transgenic (non‐Tg) and Tg *W06A11.1* (c) OE animals and (d) rescued *W06A11.1(tm4056)* mutants at 37°C heat stress. Data are representatives of 2 independent replicates and comparisons were made by Mantel‐Cox log‐rank (*n* = 90–120/strain; *, *p* < 0.05; comparison of (c) wildtype and non‐Tg to Tg and (d) wildtype and Tg to non‐Tg). (e) Analysis of mitochondrial connectivity in *W06A11.1* OE Tg and non‐Tg siblings at 20°C (see Figure S8b, c for representative images and additional mitochondrial quantification). Data are representatives of 2 independent replicates (per strain; *n =* 30, number of ROIs = 157–167) and comparisons were made by Mann–Whitney for each mitochondrial feature (presented as mean ± SD; n.s, *p* ≥ 0.05; **, *p* < 0.01; ***, *p* < 0.001; ****, *p* < 0.0001). All animals were developed at 20°C to day 1 of adulthood and where indicated, were further exposed to 37°C heat stress for (a, e) 3 h, (b) 5 h, or (c, d) until death.

Since W06A11.1 is required by neuronal HLH‐30/TFEB for promoting heat stress resistance (Figure 3d, Figure S7a), we sought to determine its involvement in mitochondrial fragmentation. To this end, we compared mitochondrial morphologies in the absence and presence of the *W06A11.1(tm4056)* loss of function mutation. We found that *W06A11.1* loss of function repressed mitochondrial fragmentation during heat stress in wildtype and neuronal HLH‐30/TFEB rescued animals while having no effect in *hlh‐30(tm1978)* mutants, strongly supporting that induction of mitochondrial fragmentation by neuronal HLH‐30/TFEB for heat stress resistance is W06A11.1‐dependent (Figure 5a, Figure S7b–d).

To further characterize the role of W06A11.1 in fragmenting mitochondrial during heat stress, we drove W06A11.1::DsRed overexpression under its own promoter in wildtype animals. Although we hypothesized that it functions in muscles to mediate heat stress resistance, we surprisingly observed its expression in a few pharyngeal‐surrounding cells in the head, possibly of the nervous system due to their enrichment and similar spatial organization in the head (White et al., 1986), while having no detectable expression in muscles (Figure 5b). Notably, W06A11.1 expression in these cells was not uniformly observed in all animals at control temperature but robustly increased following heat stress, indicating that W06A11.1 expression is heat stress inducible (Figure 5b). Additionally, we confirmed that W06A11.1 expression is HLH‐30/TFEB‐dependent as the reporter was not upregulated by heat stress in *hlh‐30(tm1978)* mutants (Figure S8a). Importantly, W06A11.1 overexpression improved survival at heat stress (Figure 5c, Figure S8b) while rescue of W06A11.1 restored the compromised survival of *W06A11.1(tm4056)* mutants to wildtype levels (Figure 5d). Lastly, W06A11.1 overexpression was sufficient to enhance mitochondrial fragmentation in the muscles despite undetectable expression in this tissue (Figure 5e, Figure S8c, d). Taken together, these findings strongly demonstrate that W06A11.1 is a bona fide mediator of thermoresistance by inducing peripheral mitochondrial fragmentation.

### 2.6 Neuronal HLH‐30/TFEB induces peripheral mitochondrial fragmentation by modulating neurotransmission to mediate thermoresistance

Since neuronal HLH‐30/TFEB induces peripheral mitochondrial fragmentation during heat stress, we hypothesized that this occurs through its regulation of neurotransmission or neurosecretion signaling events. To test this, we leveraged the *unc‐13(e1091)* and *unc‐31(e928)* loss of function mutations which result in defective synaptic vesicle and dense core vesicle (DCV) release, respectively (Richmond et al., 1999; Speese et al., 2007). We found that defective DCV release universally improved the thermoresistance of all three genotypes (Figure S9a), demonstrating that neurosecretory events are HLH‐30/TFEB‐independent and detrimental for heat stress resistance, in line with a previous study showing a deleterious effect of neurosecretory signaling on proteostasis in the muscle (Prahlad & Morimoto, 2011). Additionally, mitochondrial fragmentation was not affected by defective DCV release, indicating that antagonism of heat stress resistance by neurosecretory signaling occurs via a separate mechanism (Figure S9b–d).

**FIGURE 6:**
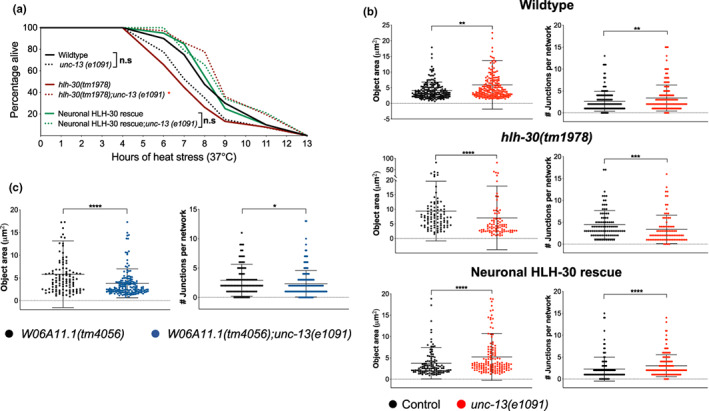
Neuronal HLH‐30 induces peripheral muscle mitochondria fragmentation by modulating neurotransmission during heat stress. (a) Survival analyses of wildtype, *hlh‐30(tm1978)*, and neuronal HLH‐30/TFEB rescued animals in the absence (solid lines) and presence (dotted lines) of *unc‐13(e1091)* loss of function at 37°C heat stress. Data are representative of 3–4 independent replicates (*n* = 115–170/strain) and comparisons were made by Mantel‐Cox log‐rank (n.s, *p* ≥ 0.05; *, *p* < 0.05; comparisons of *unc‐13(e1091)* to control per genotype). (b) Analysis of mitochondrial connectivity in the presence and absence of *unc‐13(e1091)* loss of function in wildtype, *hlh‐30(tm1978)*, and neuronal HLH‐30/TFEB rescued animals after 37°C heat stress for 3 h (see Figure S10 for additional mitochondrial quantification). (c) Comparison of mitochondrial connectivity between *W06A11.1(tm4056)* and *W06A11.1(tm4056);unc‐13(e1091)* animals after 37°C heat stress for 3 hrs (see Figure S11 for additional mitochondrial quantification). Data are representatives of (b) 3–5 independent replicates (per strain; *n* = 30–50; number of ROIs = 87–196) and (c) 2–3 independent replicates (per strain; *n* = 30; number of ROIs = 107–161) and comparisons were made by Mann–Whitney for each mitochondrial feature (presented as mean ± SD; n.s, *p* ≥ 0.05; *, *p* < 0.05; **, *p* < 0.01; ***, *p* < 0.001; ****, *p* < 0.0001). All animals were developed at 20°C to day 1 of adulthood and were further exposed to 37°C heat stress (a) until death or for (b, c) for 3 h.

In contrast, defective synaptic vesicle release markedly improved the thermoresistance of *hlh‐30(tm1978)* mutants (Figure 6a), which corresponded with enhanced heat stress‐induced mitochondrial fragmentation in muscles (Figure 6b, Figure S10b). Notably, loss of neurotransmission impacted mitochondrial fragmentation in wildtype and neuronal HLH‐30/TFEB rescued animals although thermoresistance was not significantly affected, suggesting that there could be additional mechanisms employed by neuronal HLH‐30/TFEB to mediate heat stress resistance, which are separate from neurotransmission‐dependent peripheral mitochondrial fragmentation (Figure 6b, Figure S10a, c). Overall, these findings suggest that neurotransmission signaling impairs thermoresistance and that neuronal HLH‐30/TFEB may promote thermoresistance by preventing excessive neurotransmission.

Since we observed possible W06A11.1 expression in the nervous system (Figure 5b), we next sought to determine if W06A11.1 may induce peripheral mitochondrial fragmentation during heat stress by regulating neurotransmission. We found that *W06A11.1(tm4056);unc‐13(e1091)* mutants exhibited a significantly greater extent of mitochondrial fragmentation during heat stress than *W06A11.1(tm4056)* animals, suggesting that W06A11.1 regulates neurotransmission to influence peripheral mitochondrial fragmentation (Figure 6c, Figure S11). Taken together, our findings suggest that neuronal HLH‐30/TFEB modulates neurotransmission signaling events dependently on W06A11.1 to regulate mitochondrial fragmentation in peripheral tissues.

## 3 DISCUSSION

A mechanistic understanding of lifespan and stress resistance regulation by TFEB, as well as its cell‐ and non‐cell autonomous‐associated mechanisms, is crucial to properly leverage TFEB activity to promote healthy aging. Our study provides pioneering evidence that HLH‐30/TFEB in neurons regulates heat stress resistance and IIS‐related longevity by distinct mechanisms. Importantly, we report that neuronal HLH‐30/TFEB modulates neurotransmission through the hitherto uncharacterized protein W06A11.1 to stimulate mitochondrial fragmentation in peripheral tissues, in turn mediating systemic heat stress resistance in *C. elegans*.

Previous studies have demonstrated that HLH‐30/TFEB is systemically required for maintaining normal lifespan, longevity, and heat stress resistance in *C. elegans* (Lapierre et al., 2013; Lin et al., 2018; Visvikis et al., 2014). As neurons are important for integrating signaling events to regulate these events (Miller et al., 2020), we hypothesized that HLH‐30/TFEB activity in neurons may be similarly important for upkeeping these processes. Notably, we observed that neuronal HLH‐30/TFEB is essential for IIS‐dependent longevity but redundant for thermoresistance in long‐lived *daf‐2* mutants. Conversely, it is not necessarily required for normal lifespan but important for thermoresistance in nonlong‐lived animals. These diametrically opposite requirements of neuronal HLH‐30/TFEB for lifespan and heat stress resistance in longevity‐promoting and nonpromoting genetic backgrounds highlight a potential divergence of these regulatory mechanisms. Indeed, they were uncoupled with DAF‐16/FOXO, which was required by neuronal HLH‐30/TFEB for the promotion of longevity but not for thermoresistance. This corroborated separate findings of context‐dependent synergy between HLH‐30/TFEB and DAF‐16/FOXO for the regulation of longevity but not heat stress resistance (Lin et al., 2018). Notably, previous work has shown that the neuronal activity of another transcription factor, HSF‐1, can also employ separate mechanisms to regulate longevity and heat stress resistance (Douglas et al., 2015). Hence, our findings provide further compelling evidence that transcription factors can employ context‐specific mechanisms to regulate systemic processes from neurons. Additionally, our observations support that the association of enhanced stress resistance with longevity is largely contextual (Dues et al., 2017, 2019). Of note, we performed analyses at 25°C so that lifespan benefits dependent on neuronal HLH‐30/TFEB rescue could be discriminated since the reduced survival of *hlh‐30(tm1978)* mutants was only detectable at 25°C (Lapierre et al., 2013; Lin et al., 2018; Visvikis et al., 2014). To maintain consistency, we further investigated lifespan for long‐lived *daf‐2* mutants at the same temperature. However, a caveat to acknowledge is that animals were under chronic mild heat stress at 25°C which may occlude genetic and mechanistic differences between lifespan and thermoresistance to be fully elucidated. Taken together, this places emphasis on the importance of dissecting the diverse tissue‐specific mechanisms of longevity‐ and stress‐modulating proteins such as HLH‐30/TFEB to better target age‐associated decline.

Transcriptional responses to heat stress, such as HSF1‐dependent upregulation of the heat shock response, are important in enabling organisms to counteract heat‐associated proteostatic insults (Morimoto, 2011; Rodriguez et al., 2013). Herein, we identified heat‐induced, neuronal HLH‐30/TFEB‐dependent transcriptional changes which were similarly preserved in the presence of ubiquitous HLH‐30/TFEB, suggesting that neuronal HLH‐30/TFEB markedly affects peripheral gene expression and/or processes to bring about detectable transcriptional changes at the systemic level. We surprisingly found a downregulation of *hlh‐30/TFEB* transcripts with heat stress which was not attributed to reduced nuclear entry (Figure 2a, Figure S3a, b and S5b), suggesting possible negative autoregulation which is reported to be important for speeding transcriptional responses (Rosenfeld et al., 2002). Most importantly from our transcriptional analyses, we subsequently confirmed that W06A11.1, which was transcriptionally induced dependently on neuronal HLH‐30/TFEB during heat stress, was functionally required by neuronal HLH‐30/TFEB for neurotransmission regulation to promote systemic heat stress resistance through the induction of peripheral mitochondrial fragmentation. We unexpectedly observed W06A11.1 expression in the head instead of the muscle as we initially hypothesized, and these observations raise an important question of the exact identity of these cells. As the head of *C. elegans* is enriched with cells of the nervous system (White et al., 1986), these cells are possibly neuronal or glial subsets. We speculate them to be the former since we observed that W06A11.1 regulates neurotransmission signaling to influence mitochondrial fragmentation in the periphery. It is unclear how neuronal HLH‐30/TFEB regulates W06A11.1 to modulate neurotransmission for inducing peripheral mitochondrial fragmentation, but observations herein suggest that hyperactivated neuronal signaling to the periphery impairs heat stress resistance. Notably, imbalanced neuronal hyperstimulation due to excessive cholinergic excitation and loss of gamma‐aminobutyric acid inhibition at neuromuscular junctions was shown to be detrimental for proteostasis in muscles (Garcia et al., 2007), suggesting that excessive neuronal signaling can be similarly inconducive for counteracting heat‐induced proteostatic insults. However, we did not observe enrichments of proteostasis‐related genes which were dependent on neuronal HLH‐30/TFEB (Figures S4a, b and S5a), suggesting the involvement of proteostasis‐independent processes. However, it should be noted that we only analyzed transcriptional changes at a single time point, and it is possible that proteostasis‐related genes may be transcriptionally induced by neuronal HLH‐30/TFEB at either longer durations of heat stress or during recovery from it. As proteostatic processes such as autophagy were shown to be important for the hormetic effects of heat stress following removal from stress (Chen et al., 2021; Kumsta et al., 2017), these additional analyses are important to address in future studies to understand if neuronal HLH‐30/TFEB can promote healthy aging after stress exposure. Furthermore, it will be important to elucidate the neuromodulatory mechanisms which are dependent on neuronal HLH‐30/TFEB to further understand the mechanistic processes leading to peripheral mitochondrial fragmentation.

An unexpected finding of our study is the requirement of mitochondrial fragmentation against heat stress as mitochondrial fragmentation was previously shown to be detrimental for longevity (Burkewitz et al., 2015; Gerisch et al., 2020; Zhang et al., 2019; Zhou et al., 2019). Of note, previous observations associating adult reproductive diapause‐induced longevity with HLH‐30/TFEB‐dependent inhibition of muscle mitochondrial fragmentation (Gerisch et al., 2020) demonstrated a reverse role of HLH‐30/TFEB in promoting mitochondrial fusion rather than fragmentation to mediate longevity, which suggests context‐specific mitochondrial dynamics regulation by HLH‐30/TFEB. Interestingly, a separate study also showed that HLH‐30/TFEB can also be conversely activated by mitochondrial dynamics disruption to regulate lifespan (Liu et al., 2020), raising the possibility that HLH‐30/TFEB can be cross regulated by mitochondrial fragmentation in addition to regulating it to promote heat stress protection. An intriguing observation herein was that *drp‐1* knockdown compromised the thermoresistance of wildtype but not of neuronal HLH‐30/TFEB animals. As we have observed lower food consumption from neuronal HLH‐30/TFEB rescued animals than wildtype controls, it is possible that higher residual *drp‐1* mRNA levels due to reduced RNAi ingestion and gene knockdown may adequately induce mitochondrial fragmentation for maintaining thermoresistance in these animals. However, as knockdowns of other fission genes (*fis1*, *fis‐2*, *mff‐1*, and *mff‐2*) had deleterious effects on thermoresistance, additional differences in endogenous expression levels and knockdown efficiencies of the double‐stranded RNA between these genes may also contribute toward the phenotypic disparities observed herein. Overall, we have demonstrated through a panel of fission genes that mitochondrial fragmentation was functionally important for neuronal HLH‐30/TFEB‐dependent thermoresistance.

An important question to address is how heat stress resistance is mediated through mitochondrial fragmentation. Although mitochondrial fragmentation is a precursor to mitophagy (Ni et al., 2015) which may be thermoprotective (Chen et al., 2021), the lack of enrichment of autophagy‐related genes with heat stress in our transcriptomics analyses suggest that mitochondrial fragmentation may not necessarily mediate heat stress resistance through mitophagic events. As discussed above, these events may be important in the recovery phase following heat stress exposure rather than during stress per se. Another possibility is that heat stress protection may be mediated by mitochondrial signals which are regulated by mitochondrial dynamics, such as signaling through reactive oxygen species (Chandel, 2015; Labbé et al., 2014). Notably, mitochondrial fragmentation induced by cell injury was shown to be important for mediating localized plasma membrane repair through mitochondrial calcium‐dependent redox signaling, exemplifying a protective role of mitochondrial fragmentation for stress mitigation (Horn et al., 2020). Altogether, our work revealed a novel non‐cell autonomous mechanism of thermoresistance originating in neurons, and puts neuronal HLH‐30/TFEB as an important target of interest to promote systemic stress resistance and healthy aging.

## 4 METHODS

### 4.1 *C. elegans* strains

All strains were cultured on *Escherichia coli* OP50 on nematode growth media (NGM) agar at 20°C as previously described (Brenner, 1974; Stiernagle, 2006) (additional strain details provided in Table S4). Synchronous populations were obtained either by sodium hypochlorite bleaching or egg picking.

### 4.2 Generation of HLH‐30::GFP and W06A11.1::DsRed DNA constructs

The DNA construct pLP27 (*rab‐3p::hlh‐30::GFP::3XFLAG::rab‐3)* was designed to neuronally restrict *hlh‐30* transgene expression with the promoter and 3′UTR regulatory elements of the neuronal *rab‐3* gene. To generate this construct, the *unc‐54* 3′UTR in pLP21 (*rab‐3p::hlh‐30::GFP::3XFLAG::unc‐54*) was replaced by the *rab‐3* 3′UTR. The following describes the sequential generation of pLP21, followed by pLP27. The 3XFLAGtag was cloned from pLP15 (Addgene plasmid #55180) with 5′ KpnI and 3′ EcoRI sites (primers LC127 and 128) into the corresponding restriction digestion sites of pLP9 (Addgene plasmid #1497, Fire Lab vector kit construct pPD95.81) to generate pLP16 (pPD95.81_*3XFLAG*). The *hlh‐30* coding sequence was then cloned from *C. elegans* complementary DNA with 5′ KpnI and 3′ NaeI sites (primers LC154 and 155) and inserted upstream of the 3XFLAG‐tag into the corresponding restriction digestion sites of pLP16. The construct was amplified (primers LC184 and 185) and inserted with the *GFP* sequence cloned from pLP19 (*hlh‐17p::GFP*) (primers LC186 and 187) between *hlh‐30* and *3XFLAG*‐tag sequences with HiFi cloning (NEBuilder® HiFi DNA Assembly Cloning Kit, New England BioLabs Inc., Ipswich, MA). Lastly, the *rab‐3* gene promoter was cloned from pLP17 (*rab‐3p::sid‐1*) with 5′ SphI and 3′ KpnI sites (primers LC143 and 188) into the corresponding restriction digestion sites of the preceding construct to generate pLP21. To derive pLP27 from pLP21, the *rab‐3* gene 3′UTR was amplified from *C. elegans* genomic DNA (primers LC227 and 228) and inserted in place of *unc‐54* 3′UTR in pLP21 (amplified with primers LC225 and 226) through HiFi cloning. pLp17 (plasmid #462) and pLP19 (plasmid #836) were kindly provided by Dr. Andrew Dillin (UC Berkeley, CA).

The DNA construct pLP30 (*W06A11.1p::W06A11.1::DsRed::unc‐54)* was designed to drive *W06A11.1* transgene expression under its promoter. pLP30 was generated by replacing the *p62* promoter and coding sequences of pLP26 (*p62p::p62::dsRED::unc‐54*) (amplified with primers LC341 and LC342) with the *W06A11.1* promoter (amplified from *C. elegans* genomic DNA with primers LC353 and 354) and coding sequence (amplified from *C. elegans* complementary DNA with primers LC345 and 346) through HiFi cloning (NEBuilder® HiFi DNA Assembly Cloning Kit, New England BioLabs Inc., Ipswich, MA).

Primers used are listed in Table S5.

### 4.3 Construction of transgenic strains

Transgenic strains expressing *W06A11.1::DsRed* were generated by microinjecting the germlines of day 1 wildtype N2 adults with 25 ng/μL of pLP30 (*W06A11.1p::W06A11.1::DsRed::unc‐54* 3′UTR*)* with 5 ng/μL of pLP7 (*myo‐2::GFP*) as a selection marker. Transgenic strains expressing ubiquitous or neuronal *hlh‐30::GFP* were generated by microinjecting the germlines of day 1 wildtype N2 or *hlh‐30(tm1978)* adults with 10 ng/μl of pLp11 (*hlh‐30p::hlh‐30::GFP::unc‐54*) (Lapierre et al., 2013) or 25 ng/μl of pLP27 (*rab‐3p::hlh‐30::GFP::3XFLAG::rab‐3* 3′UTR), respectively, with 85 ng/μl of pLP24 (*unc‐122p::RFP)* as a selection marker. pLP7 and pLP24 were obtained from Addgene as plasmids #26347 (Semple et al., 2010) and #8938 (Miyabayashi et al., 1999), respectively. Where indicated in Table S4, transgenic progenies were integrated for extrachromosomal arrays by UV irradiation, and further outcrossed to wildtype animals for 10 times. The expression and nuclear localization of HLH‐30::GFP were visualized by imaging animals immobilized onto NGM agar with 0.1% sodium azide with a Zeiss Discovery V20 fluorescence microscope (Zeiss, White Plains, NY). To prevent nuclear localization caused by stress from immobilization, imaging was performed within 5 mins of mounting (Lapierre et al., 2013).

### 4.4 Genotyping

Individual animals were lysed in 10X Standard Taq buffer/proteinase K, and lysates were used as genomic DNA templates for PCR amplification with OneTaq® Quick‐Load® 2X Master Mix with Standard Buffer (all materials from New England BioLabs, Ipswich, MA). Genotyping was performed with primers listed in Table S5 with the following thermocycling conditions: Initial denaturation at 95°C (5 mins), 40 cycles of denaturation (95°C, 45 s)/annealing (60°C, 45 s)/extension (72°C, 1 min/kb product), and final extension at 72°C (10 min).

### 4.5 Lifespan analysis

Synchronous populations were developed at 20°C on OP50 until day 1 of adulthood, followed by growth at 25°C on plates containing either OP50 or RNAi clones in HT115 *Escherichia coli* from the Ahringer library (Kamath et al., 2003). Animals were assessed for their survival every 2–3 days as previously described (Hamilton et al., 2005).

### 4.6 Analysis of survival to heat stress

Synchronized populations were developed at 20°C were shifted to 37°C for heat stress induction at day 1 of adulthood. For RNAi‐mediated knockdown of genes, animals were transferred onto RNAi clones from the Ahringer library (Kamath et al., 2003) from where indicated, either the L4 larval stage or day 1 of adulthood and maintained at 20°C for 48 h, followed by subsequent exposure to 37°C heat stress. Starting from the 3rd or 4th hour of heat stress, the survival of animals was scored every 1–3 h until all animals were dead.

### 4.7 RNA sample preparation, quantitative PCR (qPCR), and RNAseq

Approximately 10,000 to 12,000 synchronized animals developed at 20°C per independent replicate for a total of 3 or 4 replicates for qPCR and RNAseq, respectively, were washed and where indicated, either kept at control conditions (20°C) or shifted to 37°C for a nonlethal duration of 3 h on day 1 of adulthood. Animals were frozen down at −80°C and subsequently extracted for RNA as previously described (Mills et al., 2019), followed by DNase treatment with the Qiagen Rnase‐Free Dnase Set and purification by the Qiagen Rneasy Mini Kit (Qiagen, Germantown, MD). For qPCR, 1 μg of RNA per sample was reverse transcribed to complementary DNA with iScript™ Reverse Transcription Supermix for RT‐PCR (Bio‐Rad, Hercules, CA) according to manufacturer's instructions, followed by qPCR analysis of transcript levels as previously described (Lapierre et al., 2013; Mills et al., 2019). qPCR primers are listed in Table S5. For RNAseq, RNA samples were confirmed for their quality with the Agilent 2100 Bioanalyzer (Agilent Technologies, Santa Clara, CA), and sequenced by GENEWIZ (Azenta Life Sciences, South Plainfield, NJ). Briefly, RNAseq libraries were prepared with the NEBNext Ultra II RNA Library Prep Kit for Illumina (New England BioLabs Inc., Ipswich, MA) and sequenced with the Illumina HiSeq instrument (Illumina Inc., San Diego, CA) with a 2 × 150 bp Paired End configuration. Generated raw sequence files were converted into fastq data, followed by de‐multiplexing with the bcl2fastq 2.17 software from Illumina.

### 4.8 RNAseq data analysis

RNAseq outputs were further subjected to differential expression analysis by GENEWIZ. Briefly, sequence reads trimmed with Trimmomatic v.0.36 (Bolger et al., 2014) were mapped to the *C. elegans* ENSEMBL reference genome with STAR aligner v.2.5.2b (Dobin et al., 2013), followed by the quantification of gene counts from the Subread package v.1.5.2. Raw gene hit count tables were filtered to remove low and nonexpressing genes across all samples (average of <10 counts per gene across all samples). Within each genotype, pairwise comparisons of gene expression between control and heat stressed samples were subsequently performed to identify differential gene expressions induced by heat stress using DESeq2 (Love et al., 2014). Differential expression was performed using the Wald test to calculate Log_2_ fold changes and adjusted *p*‐values were generated using Benjamini & Hochberg with an alpha = 0.05. Genes with differential expressions (differentially expressed genes, DEGs) of adjusted *p‐*values <0.05 were considered significant.

Commonalities in these heat stress‐induced differential gene expressions between wildtype and neuronal HLH‐30 rescued animals that occur relative to *hlh‐30(tm1978)* mutants were further analyzed by overlapping heat stress‐induced DEGs identified from each genotype with Venny (publicly available at http://bioinfogp.cnb.csic.es/tools/venny/index.html). Overlapping comparisons were performed on only significant DEGs (*p*‐values < 0.05) from both wildtype and neuronal HLH‐30 rescued animals, whereas all DEGs (regardless of significance in differential expressions) from *hlh‐30(tm1978)* mutants were included for analysis. From these comparisons, heat stress‐induced DEGs unique to wildtype and neuronal HLH‐30/TFEB rescued animals were extracted. To additionally identify DEGs which exhibited greater extents of change with heat stress in both genotypes relative to *hlh‐30(tm1978)* mutants, the following Log_2_FC thresholds were additionally applied: Upregulated (wildtype and neuronal HLH‐30/TFEB (Log_2_FC > 1), *hlh‐30(tm1978)* (Log_2_FC < 1)), downregulated (wildtype and neuronal HLH‐30/TFEB (Log_2_FC < −1), *hlh‐30(tm1978)* (Log_2_FC > −1)).

Gene set enrichment analysis (GSEA) was performed in R using clusterProfiler (Yu et al., 2012) along with curated Gene Ontology Biological Processes gene sets within the Molecular Signature Database (MSigDB) were used. Ranked gene lists were compiled from unfiltered DEG tables ordered by log_2_ fold change and enrichment statistic was generated after 1000 permutations. Figures were generated using ggplot2 (Wickham, 2016).

### 4.9 Confocal imaging

Animals were immobilized with 0.1% sodium azide onto 3% agarose pads, followed by the acquisition of 2D images with Olympus FV3000 confocal laser scanning microscope (Olympus Scientific Solutions Americas Corp., Waltham, MA). For animals expressing GFP‐labeled body wall muscles (MYO‐3::GFP) or muscle mitochondria (Mito::GFP), imaging was performed with day 1 adults on OP50 from control (20°C) or heat stressed (3 h at 37°C) conditions, or day 2 adults grown on RNAi clones from the Ahringer library (Kamath et al., 2003) for 48 h at 20°C from the L4 larval stage, at control temperature (20°C) or after heat stress (5 h at 37°C). For animals expressing W06A11.1::DsRed, imaging was performed with day 1 adults on OP50 from control temperature (20°C) or after heat stress (5 hrs at 37°C).

### 4.10 Analysis of mitochondria morphology

Regions of interest (ROIs, 45 × 45 pixels) from Mito::GFP confocal images were analyzed and quantified for various mitochondrial parameters of network connectivity with the MitoMAPR macro in FIJI (Schindelin et al., 2012) as previously described (Zhang et al., 2019). Quantified outputs provide overall insights into mitochondrial integrity and connectivity and was used in this study to compare extent of fragmentation. Analyzed features are described in more detail previously (Zhang et al., 2019).

### 4.11 Statistics

Statistical comparisons of survival for lifespan and heat stress were performed with the Mantel‐Cox log‐rank test with STATA (StataCorp, College Station, TX). Mitochondrial parameters quantified from MitoMAPR analyses were statistically compared with Kruskal–Wallis multiple comparisons test or Mann–Whitney test after normality of data distribution was determined with GraphPad Prism 9 (GraphPad Software, San Diego, CA).

## AUTHOR CONTRIBUTIONS

S.Q.W conceived the experiments, generated strains, performed imaging, lifespan, heat stress, and data analyses, and wrote the manuscript. C.J.R performed lifespan and heat stress analyses, and provided feedback on the manuscript. D.M.B provided RNAseq analyses and figures. J.M. performed heat stress analyses. L.R.L provided conceptual feedback, performed lifespan analyses and edited the manuscript.

## FUNDING INFORMATION

This work was funded by a grant from the National Institute of Health (R01 AG051810), a Talent Recruitment Award from the New Brunswick Innovation Foundation, and a Research Chair in Precision Medicine from the New Brunswick Health Research Foundation and the Jean‐Louis Lévesque Foundation to L.R.L.

## CONFLICT OF INTEREST

None declared.

## Supporting information


Appendix S1

## Data Availability

The data that support the findings of this study are available from the corresponding author upon reasonable request. Transcriptomic RNAseq datasets generated in this study are available on NCBI GEO (Accession number: GSE200383).
