# Supplementary material for: Neuronal HLH‐30/TFEB modulates peripheral mitochondrial fragmentation to improve thermoresistance in Caenorhabditis elegans
Source: Aging Cell. 2022 Nov 23;22(3):e13741. doi: 10.1111/acel.13741 (PMC10014052; doi:10.1111/acel.13741)
Supplement: Supplementary file 1 — Appendix S1 [file ACEL-22-e13741-s001.pdf]

Supplemental Figure 1

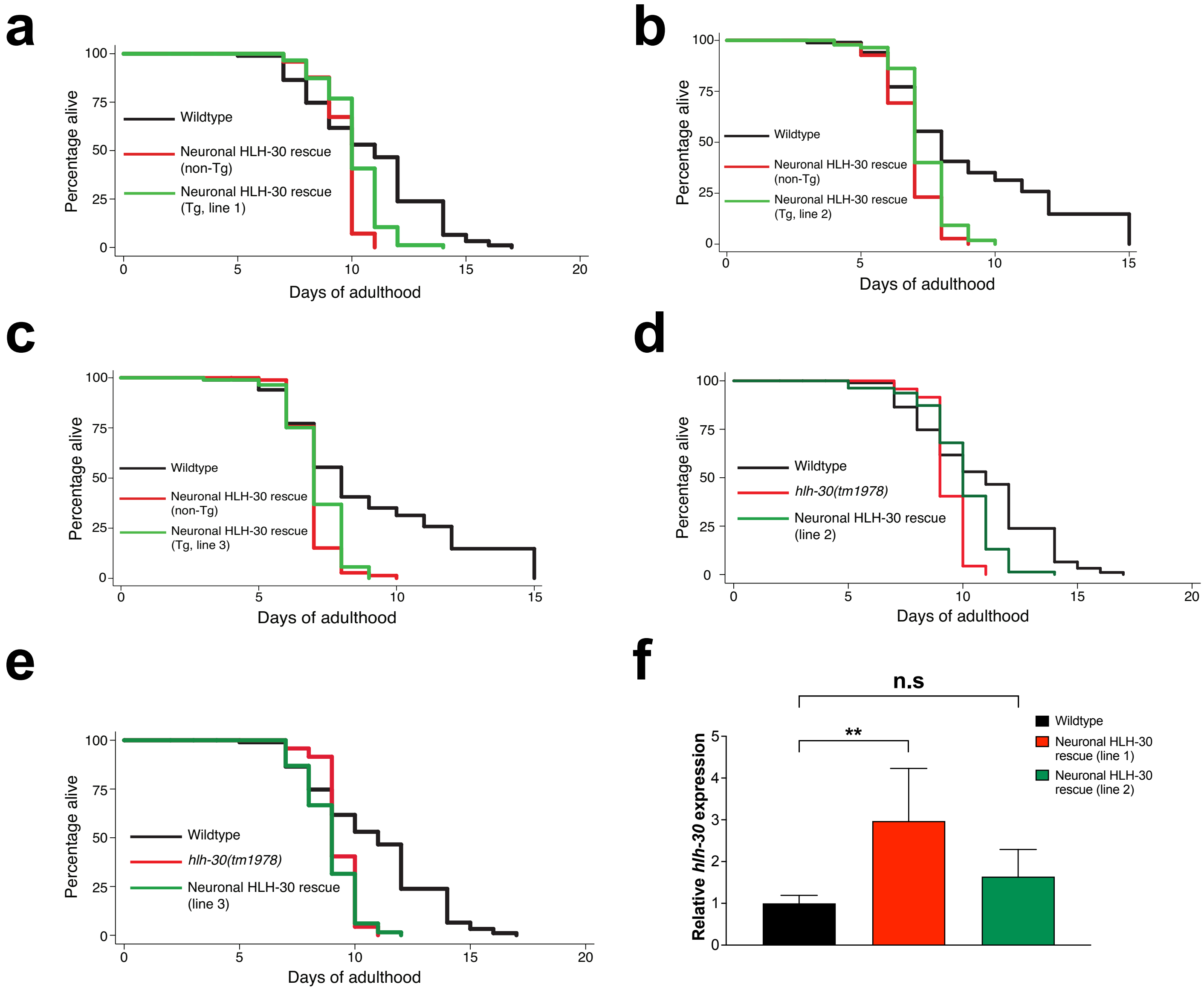

Supplemental Figure 1. Neuronal HLH-30/TFEB does not regulate normal lifespan.

Lifespan analyses of *hlh-30(tm1978)* mutants (non-transgenic (non-Tg) siblings and *hlh-30(tm1978)*) rescued with **(a to c)** extrachromosomal (Tg, transgenic) and **(d and e)** integrated arrays driving HLH-30/TFEB expression in neurons on OP50 at 25°C. Animals were developed at 20°C and shifted to 25°C on OP50 from day 1 of adulthood. Data are representatives of **(a)** 4 , **(b** and **c)** single, and (**d** and **e)** 3 independent replicates, and comparisons were made by Mantel-Cox log-rank. Further details about lifespan analyses are provided in Supplemental Table 1. **(f)** Relative *hlh-30* expression levels of wildtype and independently-derived lines 1 and 2 of neuronal HLH-30/ TFEB rescued animals which respectively exhibited absence and presence of lifespan extension in comparison to *hlh-30(tm1978)* mutants. Animals were developed at 20°C to day 1 of adulthood on OP50. Data is representative of 3 independent replicates and comparisons were made by Kruskal-Wallis (expression normalized to wildtype and presented as mean ± S.D; n.s, $p \geq 0.05$; **, $p < 0.01$)

Supplemental Figure 2

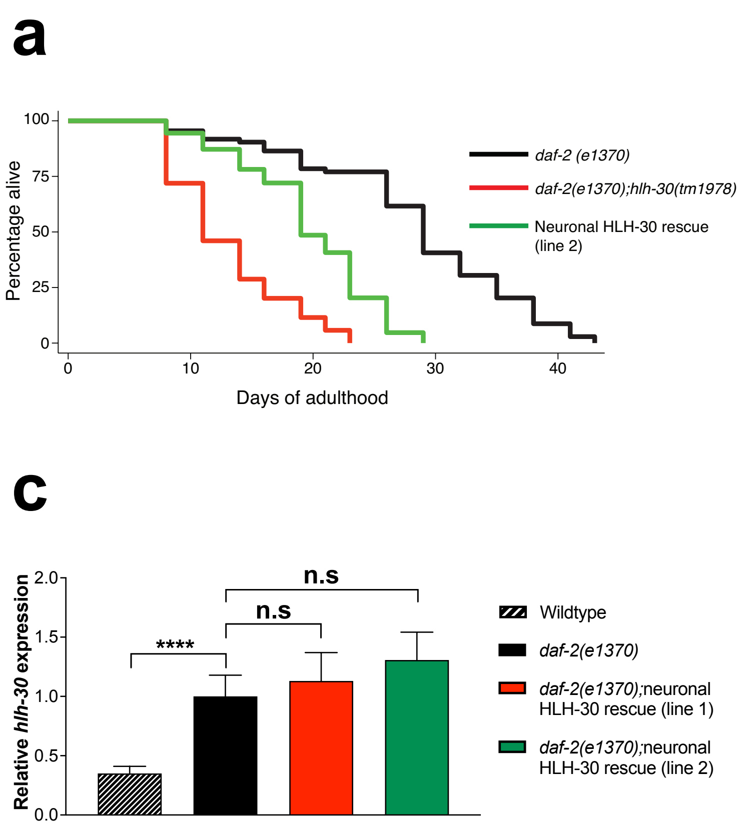

Supplemental Figure 2. Neuronal HLH-30/TFEB regulates longevity.

(a and b) Lifespan analyses of *daf-2(e1370), daf-2(e1370);hlh-30(tm1978)*, and neuronal HLH-30/TFEB rescued *daf-2(e1370);hlh-30(tm1978)* animals fed OP50 at 25°C**.** Animals were developed at 20°C and shifted to 25°C on OP50 from day 1 of adulthood. Data are representatives of 3 independent replicates, and comparisons were made by Mantel-Cox log-rank. Further details about lifespan analyses are provided in Supplemental Table 2. **(c)** Relative *hlh-30* expression levels of wildtype, *daf-2(e1370)*, and independently-derived lines 1 and 2 of neuronal HLH-30/TFEB rescued *daf-2(e1370);hlh-30(tm1978)* animals developed at 20°C to day 1 of adulthood on OP50. Data is representative of 3 independent replicates and comparisons were made by one-way ANOVA (expression normalized to *daf-2(e1370) and* presented as mean ± S.D; n.s, $p \geq 0.05$; ****, $p < 0.0001$).

Supplemental Figure 3

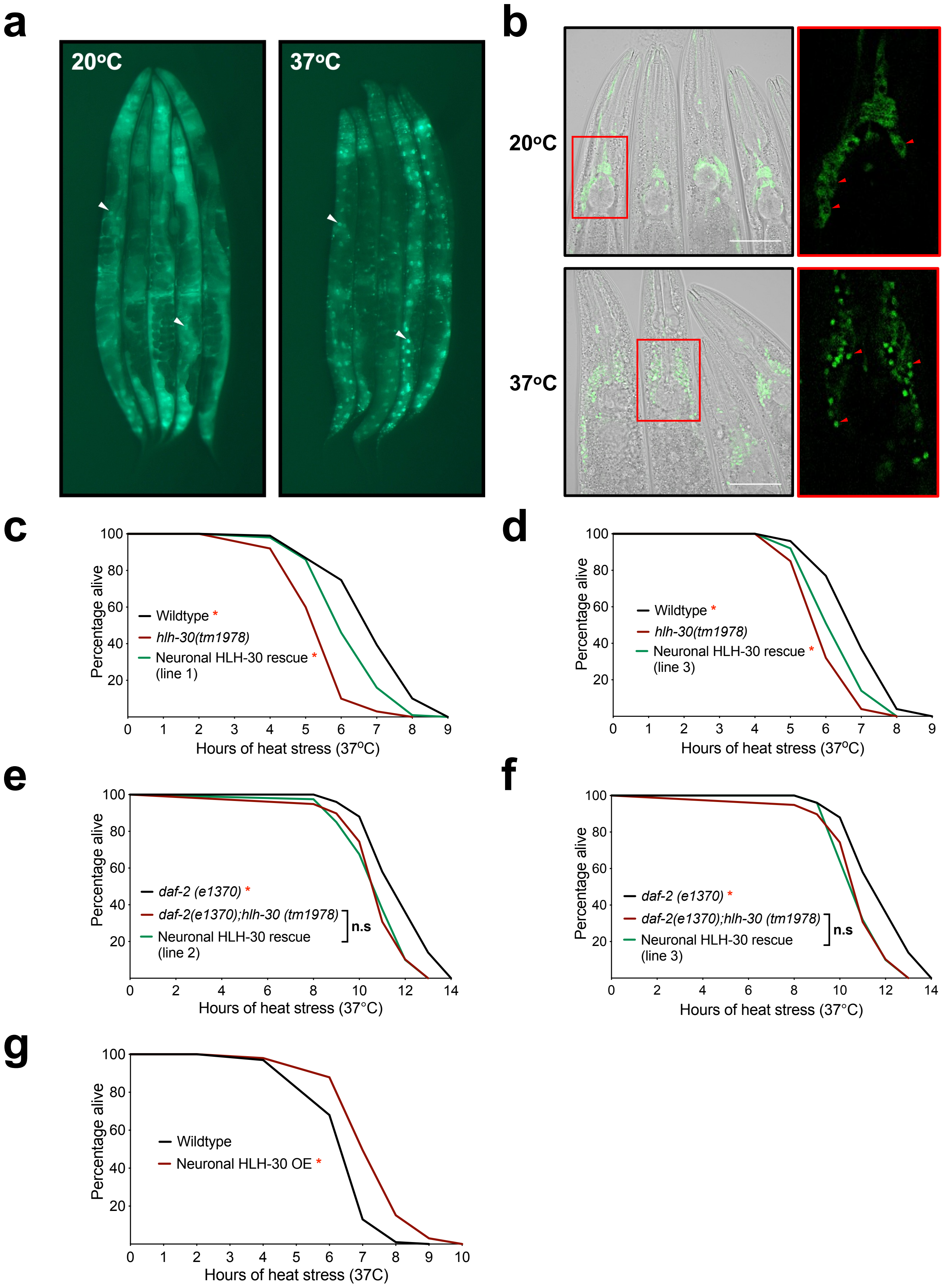

Supplemental Figure 3. Neuronal HLH-30/TFEB mediates thermoresistance in normal but not longevity-promoting conditions.

(a) *hlh-30(tm1978)* mutants ubiquitously rescued with HLH-30::GFP were developed at 20°C to day 1 of adulthood and imaged at 20°C and after 3 hours of heat stress at 37°C. Arrowheads indicate intestinal nuclear enrichment of HLH-30::GFP. **(b)** *hlh-30(tm1978)* mutants neuronally rescued with HLH-30::GFP were developed at 20°C to day 1 of adulthood and imaged for head neurons at 20°C and after 3 hours of heat stress at 37°C. Neuronal nuclei are indicated by red arrows in enlarged images on the right. Scale bars = 20 μM. Survival analyses of neuronal HLH-30/TFEB rescued animals in comparison to their **(c and d)** wildtype and *hlh-30(tm1978)* and **(e and f)** *daf-2(e1370* and *daf-2(e1370);hlh-30(tm1978)* controls at 37°C heat stress. **(g)** Survival analyses of neuronal HLH-30/TFEB overexpressing (OE) animals in comparison to their wildtype controls at 37°C heat stress. Animals were developed at 20°C and shifted to heat stress at 37°C on day 1 of adulthood. Data are representatives of **(c** and **d)** single experiments and **(e to g)** 2 independent replicates and comparisons were made by Mantel-Cox log-rank (n = 90-100/strain; n.s, $p \geq 0.05$; *, $p<0.05$; in comparison to **(c** and **d)** *hlh-30(tm1978)*, **(e and f)** *daf-2(e1370);hlh-30(tm1978))* or **(g)** wildtype animals.

Supplemental Figure 4

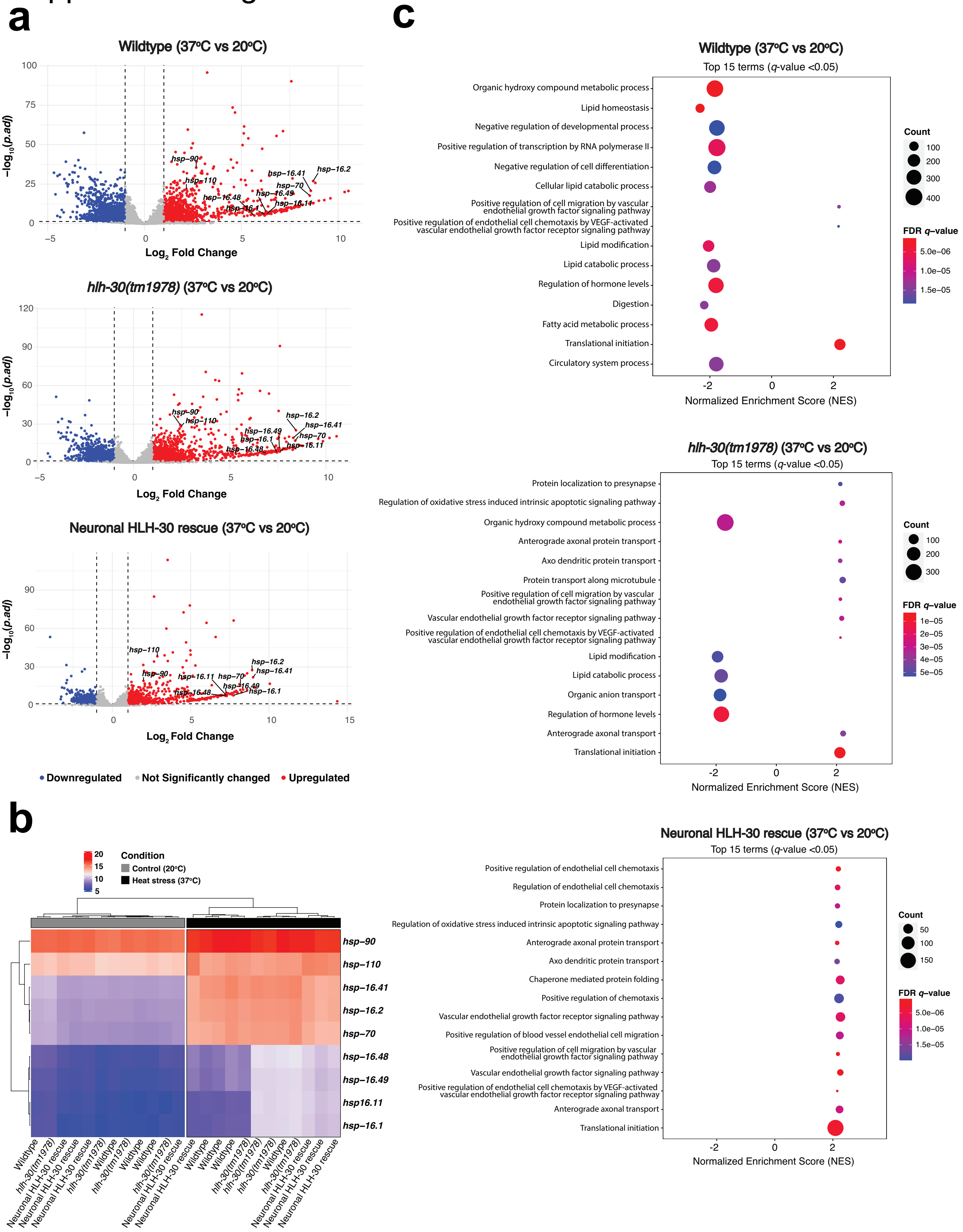

Supplemental Figure 4. Heat stress-induced transcriptional changes in wildtype, *hlh-30(tm1978)*, and neuronal HLH-30/TFEB rescued animals. **(a)** Volcano plots indicating the upregulation of heat stress-induced heat shock protein (*hsp)* genes in wildtype, *hlh-30(tm1978)*, and neuronal HLH-30/TFEB rescued animals from 37^oC (heat stress) in comparison to 20^oC (control conditions). Horizontal dotted line indicates adjusted *p*-value <0.05. Vertical dotted lines indicate >1.0 and <1.0 Log_2 fold change. **(b)** Heat map showing the regularized log transformed gene counts clustering (rlog) of animals within 20^oC (control) and 37^oC (heat stress) conditions regardless of genotypes, and the upregulation of significant (adjusted *p*-value <0.05, Log_2 fold change >1) *hsp* genes in heat stressed groups. **(c)** Gene set enrichment analysis plots of the top 15 most significantly-enriched Gene Ontology Biological Processes terms (*q* <0.05) from wildtype, *hlh-30(tm1978)*, and neuronal HLH-30/TFEB rescued animals at 37^oC (heat stress) in comparison to 20^oC (control conditions). Negative and positive normalized enrichment scores (NES) indicate respective down- and upregulation of enriched processes with heat stress. Animals from 4 independent replicates were developed at 20^oC to day 1 of adulthood and harvested for RNA after 3 hrs of further growth at 20^oC or 37^oC.

Supplemental Figure 5

a

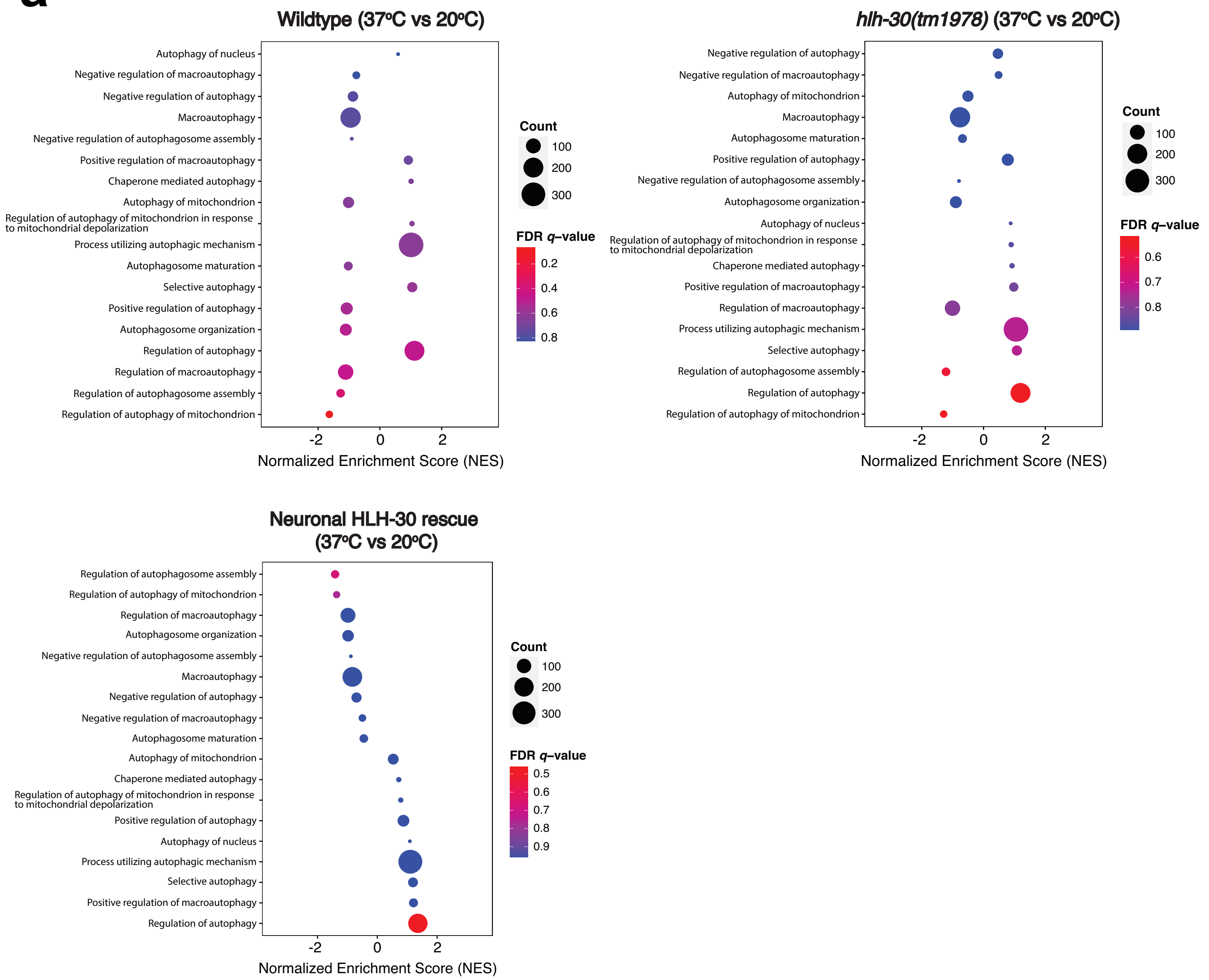

b

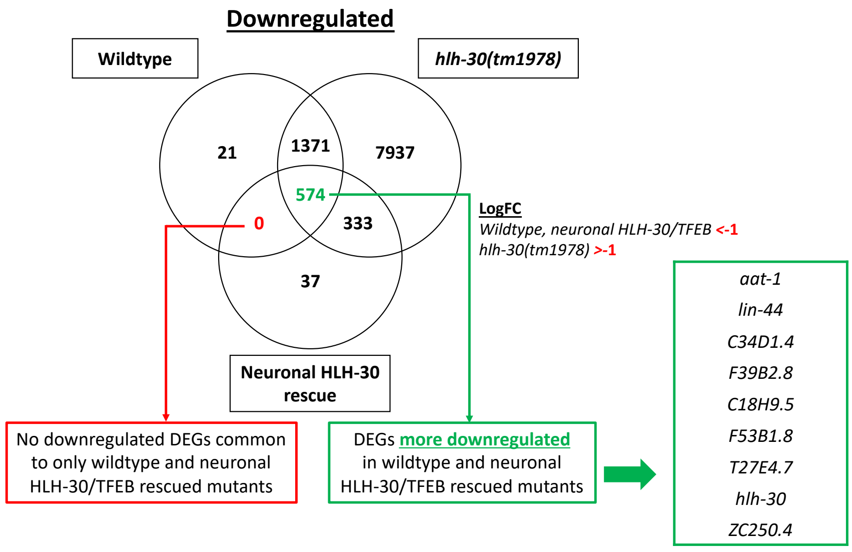

Supplemental Figure 5. Analysis of heat stress-induced autophagy-related changes and downregulated differentially expressed genes.

(a) Gene set enrichment analysis plots of Gene Ontology Biological Processes terms related to autophagy from wildtype, *hlh-30(tm1978)*, and neuronal HLH-30/TFEB rescued animals at 37°C (heat stress) in comparison to 20°C (control conditions). Note that although negative and positive normalized enrichment scores (NES) indicate respective down- and upregulation of indicated autophagic processes, none of these were significantly enriched with heat stress exposure across genotypes (q ≥0.05). **(b)** Genes downregulated by 37°C (heat stress) in comparison to 20°C (control conditions) for each genotype were overlapped to extract significant heat stress-specific differentially expressed genes (adjusted p-value <0.05) unique to or more downregulated (Log_2 fold change (LogFC) thresholds applied as indicated) in wildtype and neuronal HLH-30/TFEB rescued animals than *hlh-30(tm1978)* mutants. Animals from 4 independent replicates were developed at 20°C to day 1 of adulthood and harvested for RNA after 3 hrs of further growth at 20°C (control conditions) or 37°C (heat stress).

Supplemental Figure 6

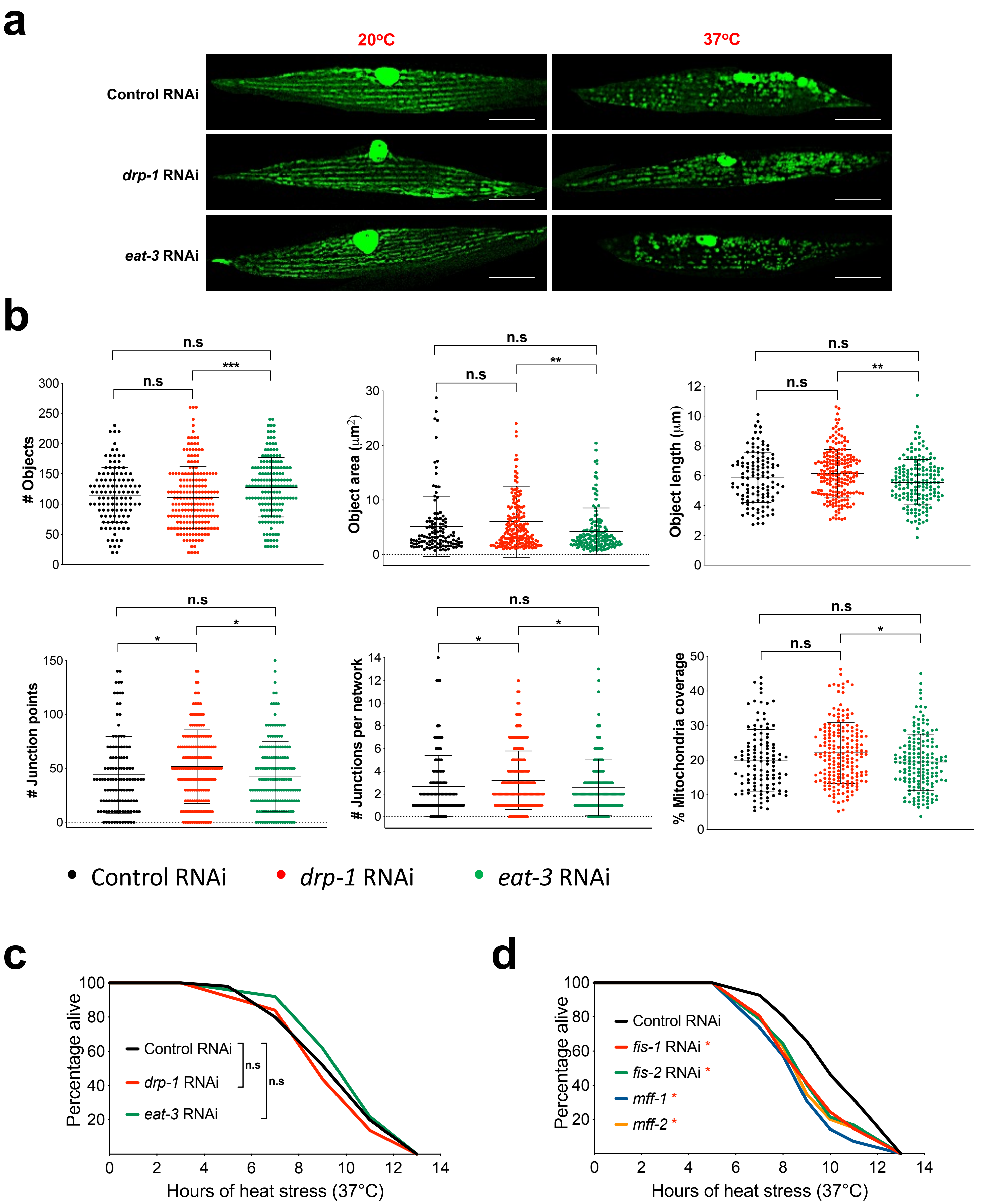

Supplemental Figure 6. Mitochondrial fragmentation is mechanistically important for thermoresistance.

(a) Representative images of muscle mitochondrial morphology with the body wall muscle mitochondrial reporter (Mito::GFP) in wildtype animals fed control RNAi (*L4440*) or RNAi against *drp-1* or *eat-3* at 20°C (control conditions) or after 5 hrs heat stress at 37°C heat stress, and **(b)** corresponding analysis of mitochondrial connectivity after heat stress. Data are representatives of 3 independent replicates (*n* = 30, number of ROIs = 118 - 193) and comparisons were made by Mann-Whitney for each mitochondrial feature (presented as mean ± S.D; n.s, $p \geq 0.05$; *, $p<0.05$; **, $p<0.01$; ***, $p<0.001$). Survival analyses of neuronal HLH-30/TFEB animals fed control RNAi (*L4440*) or RNAi against **(c)** *drp-1* and *eat-3,* or **(d)** *fis-1, fis-2, mff-1,* and *mff-2* at 37°C heat stress. Data are representatives of 2 independent replicates (**a,** *n* = 90/RNAi; **b**, *n* = 84 - 87/RNAi) and comparisons were made by Mantel-Cox log-rank (*, $p<0.05$; in comparison to control RNAi). All animals were developed at 20°C to the L4 larval stage on OP50, transferred onto bacteria expressing RNAi for 48 hrs, and exposed to 37°C heat stress for **(a and b)** 5 hrs or **(c and d)** until death.

Supplemental Figure 7

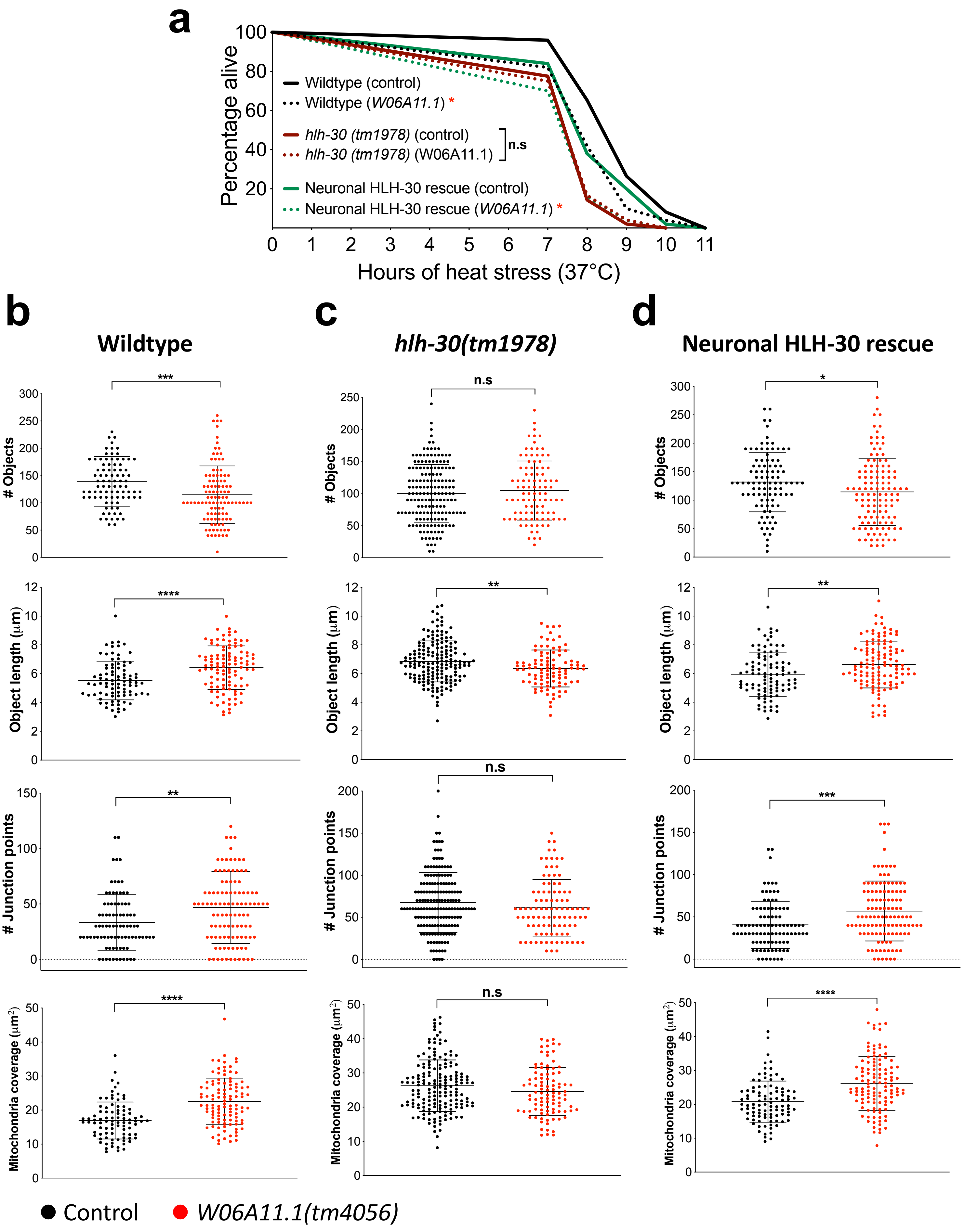

Supplemental Figure 7. Neuronal HLH-30/TFEB mediates thermoresistance through W06A11.1-dependent peripheral mitochondrial fragmentation.

(a) Survival analyses of wildtype, *hlh-30(tm1978)*, and neuronal HLH-30/TFEB rescued animals fed control RNAi (*L4440,* solid lines) or RNAi against *W06A11.1* (dotted lines) at 37°C heat stress. Animals developed at 20°C to day 1 of adulthood on OP50 were transferred onto bacteria expressing RNAi, grown at 25ºC for 48 hr, and exposed to 37°C heat stress until death. Data is representative of 2 independent replicates (n = 83 – 99/RNAi/strain) and comparisons were made by Mantel-Cox log-rank (n.s, $p \geq 0.05$; *, $p < 0.05$; in comparison to control RNAi). **(b to d)** Analysis of mitochondrial connectivity in the absence and presence of *W06A11.1(tm4056)* loss of function after 37°C heat stress for 3 hrs in **(b)** wildtype, **(c)** *hlh-30(tm1978)* loss of function mutants, and **(d)** neuronal HLH-30/TFEB rescued animals developed at 20°C to day 1 of adulthood. Data are representatives of 3 independent replicates (per strain; n = 30, number of ROIs = 88 – 171) and comparisons were made by Mann-Whitney for each mitochondrial feature (presented as mean ± S.D; n.s, $p \geq 0.05$; *, $p < 0.05$; **, $p < 0.01$; ***, $p < 0.001$; ****, $p < 0.0001$).

Supplemental Figure 8

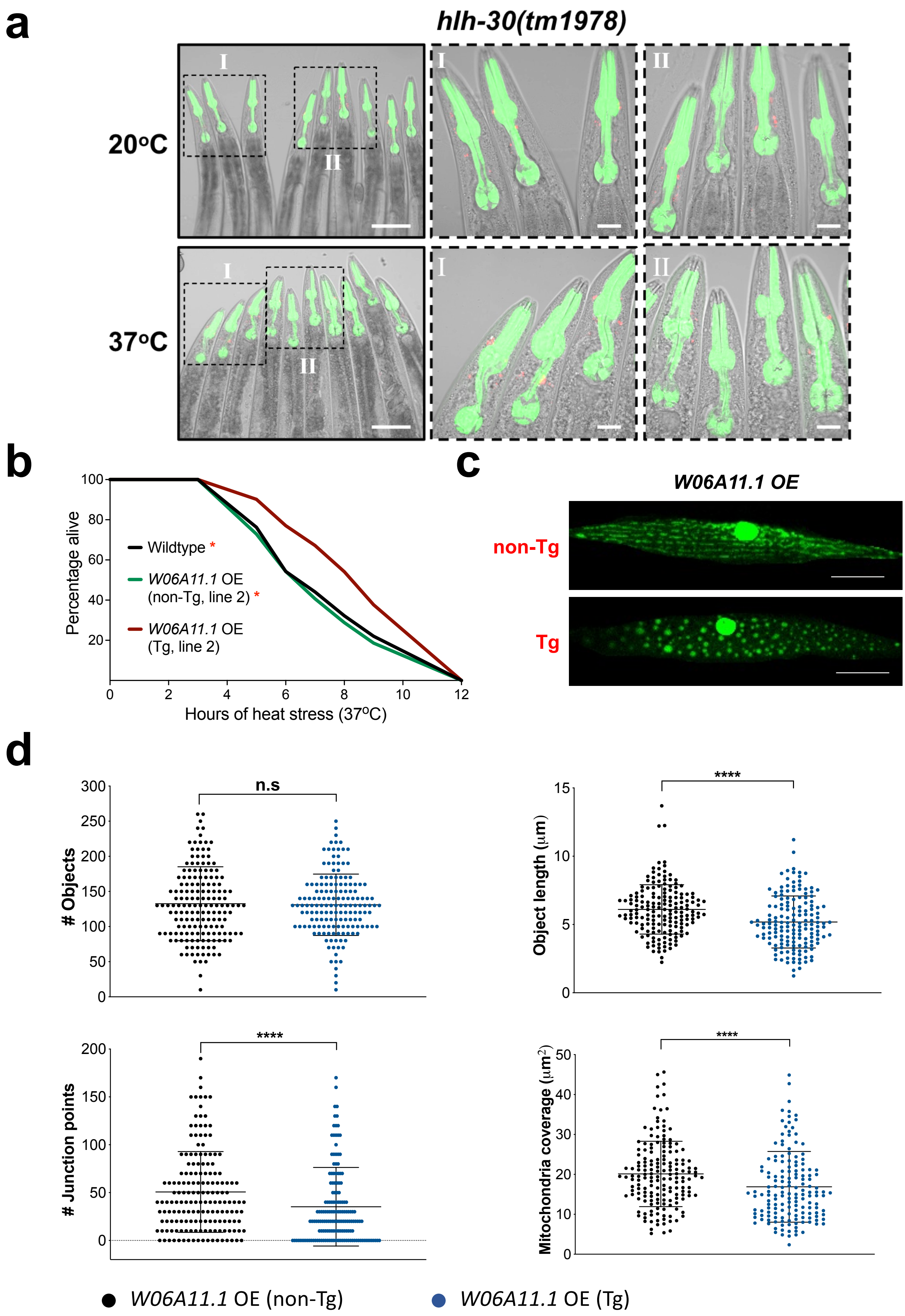

Supplemental Figure 8. W06A11.1 mediates thermoresistance through mitochondrial fragmentation.

(a) *hlh-30(tm1978)* mutants overexpressing extrachromosomal W06A11.1::DsRed at 20°C (control conditions) and after 37°C heat stress for 5 hrs. Scale bars = 100 μM, images in left column; scale bars = 20 μM, enlarged insets of head regions (I and II). **(b)** Survival analyses of wildtype, with wildtype animals overexpressing extrachromosomal W06A11.1::DsRed (*W06A11.1* OE *(Tg)*) and their non-transgenic (non-Tg) siblings at 37°C heat stress. Data is from an independent replicate and comparisons were made by Mantel-Cox log-rank (n = 60/strain; *, p<0.05; comparison of wildtype and *W06A11.1* OE (non-Tg) animals to *W06A11.1* OE (Tg)). **(c)** Representative images of muscle mitochondrial morphology with the body wall muscle mitochondrial reporter (Mito::GFP) in *W06A11.1* OE Tg and non-Tg siblings at 20°C. **(d)** Analysis of mitochondrial connectivity in *W06A11.1* OE Tg and non-Tg siblings at 20°C. Data are representatives of 2 independent replicates (per strain; n = 30, number of ROIs = 157 – 167) and comparisons were made by Mann-Whitney for each mitochondrial feature (presented as mean ± S.D; n.s, p≥0.05; ****, p<0.0001). All animals were developed at 20°C to day 1 of adulthood and where indicated, were further exposed to 37°C heat stress for **(a)** 5 hrs, **(b)** until death, or **(c and d)** 3 hrs.

Supplemental Figure 9

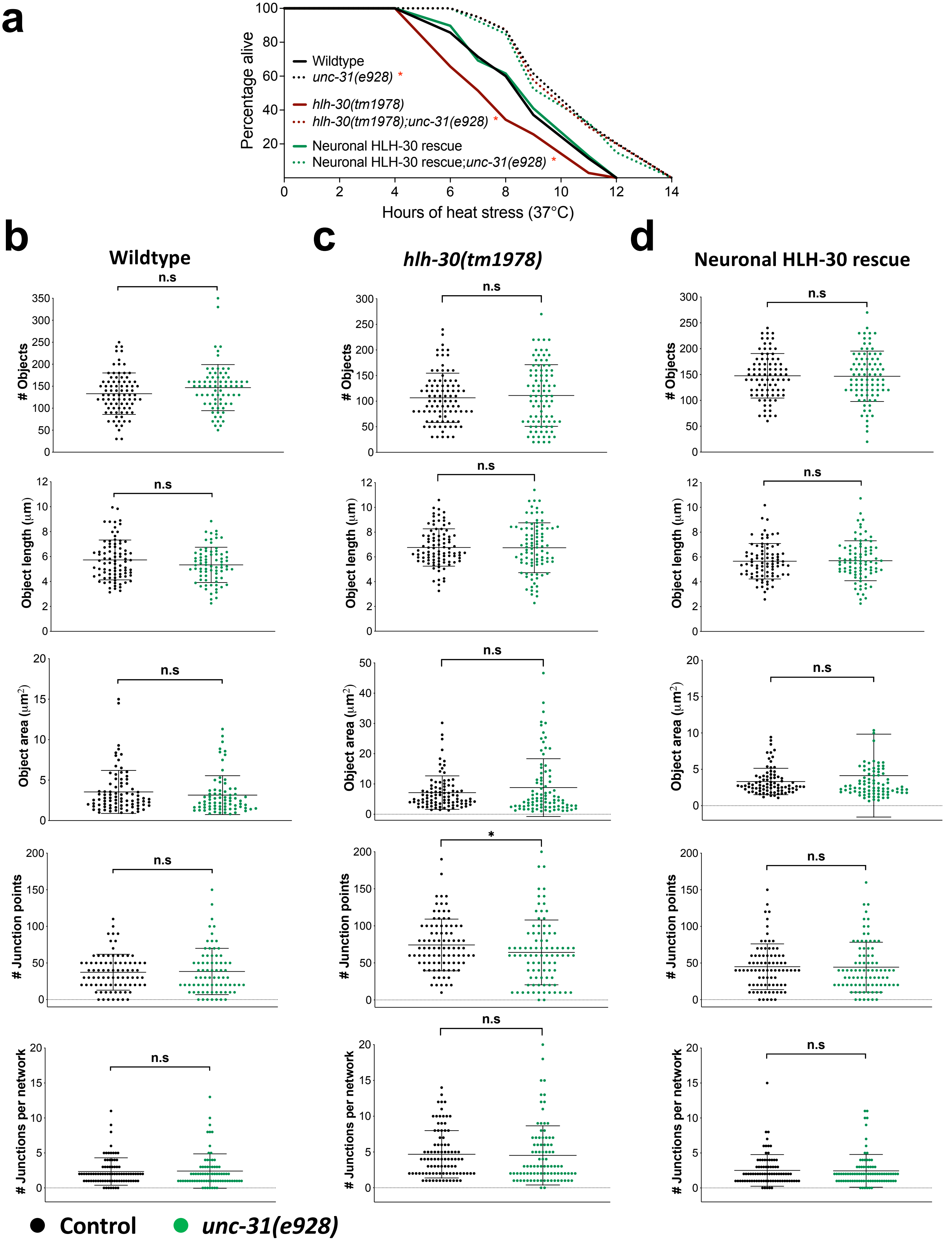

Supplemental Figure 9. Neuronal HLH-30/TFEB does not mediate mitochondrial fragmentation-dependent thermoresistance through dense core vesicle (DCV) release.

(a) Survival analyses of wildtype, *hlh-30(tm1978)*, and neuronal HLH-30/TFEB rescued animals in the absence (solid lines) and presence (dotted lines) of *unc-31(e928)* loss of function at 37°C heat stress. Animals were developed at 20°C to day 1 of adulthood and exposed to 37°C heat stress until death. Data is representative of 3 – 4 independent replicates and comparisons were made by Mantel-Cox log-rank (*n* = 113 – 169/strain; *, p<0.05; comparisons of *unc-31(e928)* to control per genotype). **(b to d)** Analysis of mitochondrial connectivity in the absence and presence of *unc-31(e928)* loss of function in **(b)** wildtype, **(c)** *hlh-30(tm1978)*, and **(d)** neuronal HLH-30/TFEB rescued animals developed at 20°C to day 1 of adulthood after 37°C heat stress for 3 hrs. Data are representatives of 3 independent replicates (per strain; *n* = 30, number of ROIs= 75 – 92) and comparisons were made by Mann-Whitney for each mitochondrial feature (presented as mean ± S.D; n.s, p≥0.05).

Supplemental Figure 10

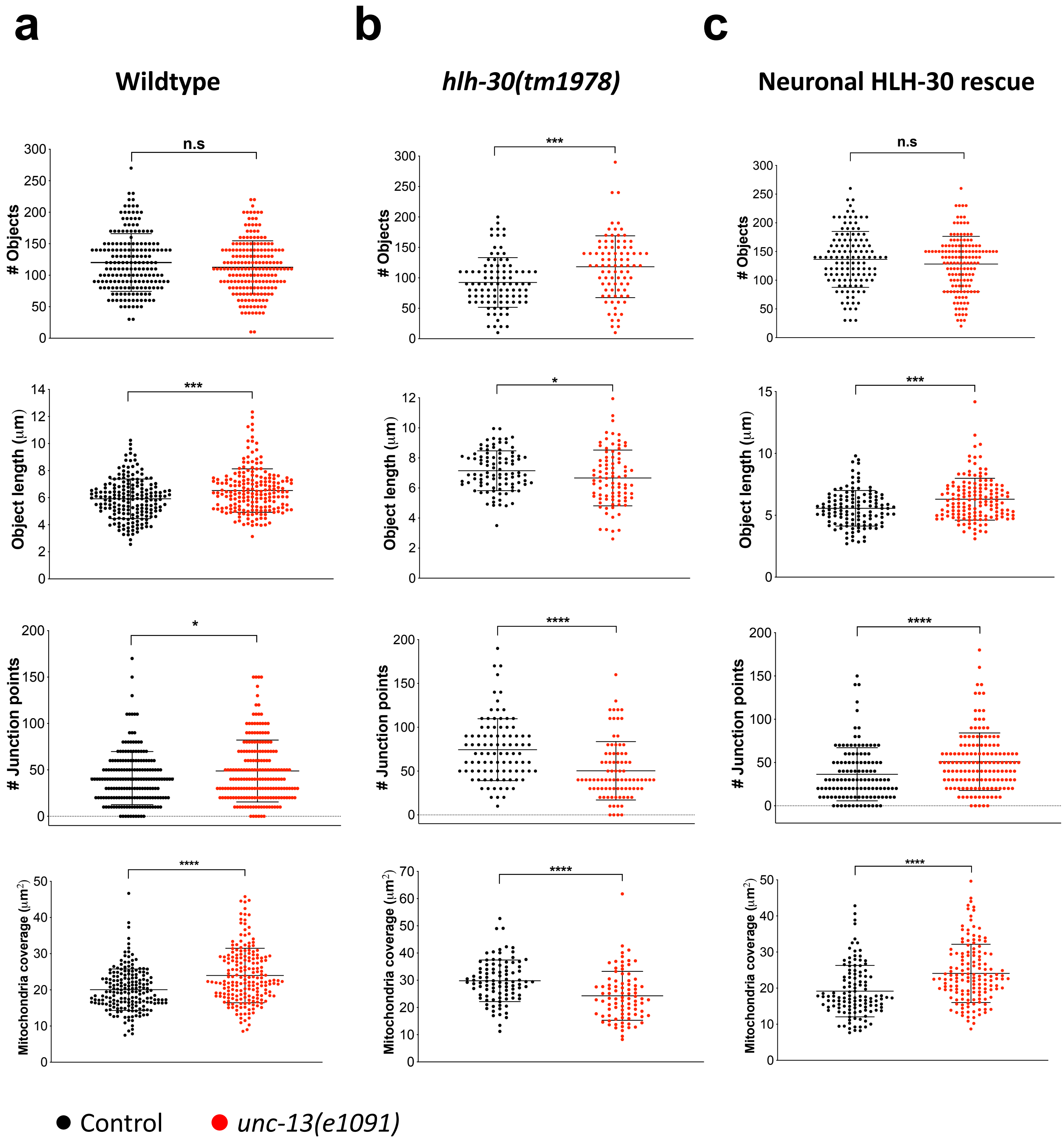

Supplemental Figure 10. Defective neurotransmission increases heat stress-induced mitochondria fragmentation in *hlh-30(tm1978)* mutants.

Analysis of mitochondrial connectivity in the absence and presence of *unc-13(e1091)* loss of function in **(a)** wildtype, **(b)** *hlh-30(tm1978)*, and **(c)** neuronal HLH-30/TFEB rescued animals developed at 20°C to day 1 of adulthood after 37°C heat stress for 3 hrs. Data are representatives of 3 - 5 independent replicates (per strain; *n* = 30 - 50; number of ROIs = 87 – 196) and comparisons were made by Mann-Whitney for each mitochondrial feature (presented as mean ± S.D; n.s, $p \geq 0.05$; *, $p<0.05$; ***, $p<0.001$; ****, $p<0.0001$).

Supplemental Figure 11

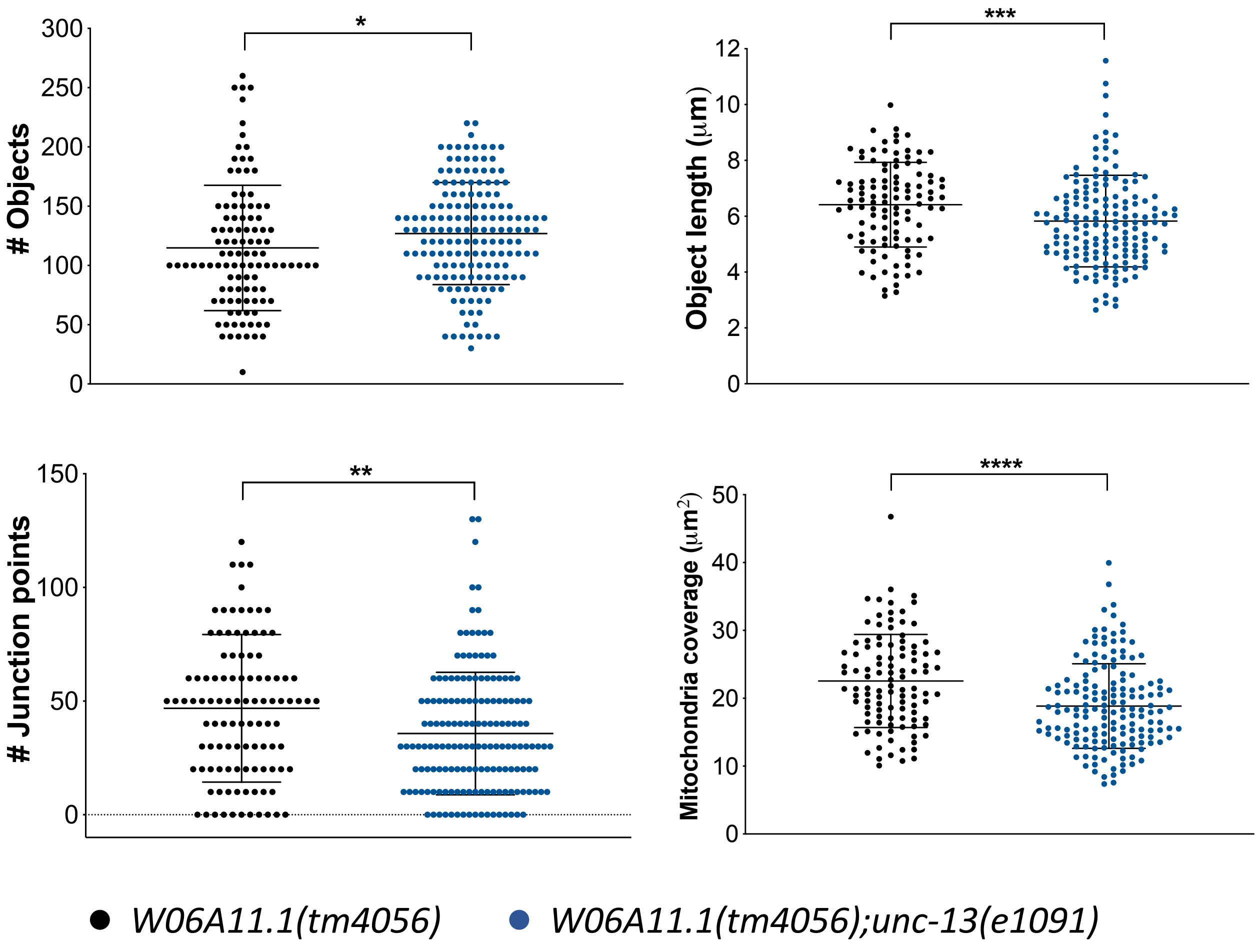

Supplemental Figure 11. W06A11.1 mediates peripheral mitochondrial fragmentation by regulating neurotransmission.

Comparison of mitochondrial connectivity between *W06A11.1(tm4056)* and *W06A11.1(tm4056);unc-13(e1091)* animals developed at 20°C to day 1 of adulthood after 37°C heat stress for 3 hrs. Data are representatives of 2 - 3 independent replicates (per strain; n = 30; number of ROIs = 107 – 161) and comparisons were made by Mann-Whitney for each mitochondrial feature (presented as mean ± S.D; n.s, $p \geq 0.05$; *, $p<0.05$; **, $p<0.01$; ***, $p<0.001$; ****, $p<0.0001$).

Supplemental Table 1

N2		*hlh-30(tm1978)*				Neuronal HLH-30 rescue strains					
**Mean LS**	**EO**	**Strain**	**Mean LS**	**EO**	**% Difference to N2**	**Strain**	**Mean LS**	**EO**	**% Difference to *hlh-30(tm1978)***	***p* Value**	**Figure #**
9.02	68	LRL81 (Non Tg)	6.9	83	-23.53	**LRL81 (Tg)**	7.34	68	6.42%	0.0012	
12.99	47	LRL81 (Non Tg)	9.57	74	-26.33%	**LRL81 (Tg)**	10.16	73	6.17%	0.0070	
10.79	93	LRL81 (Non Tg)	9.58	98	-11.21%	**LRL81 (Tg)**	10.14	86	5.85%	< 0.0001	S1a
9.48	74	LRL81 (Non Tg)	8.44	92	-0.10%	**LRL81 (Tg)**	8.96	71	6.16%	0.0086	
9.02	57	LRL82 (Non Tg)	6.86	88	-23.95%	**LRL82 (Tg)**	7.32	65	6.70%	0.0027	S1b
9.02	57	LRL83 (Non Tg)	6.94	84	-23.05%	**LRL83 (Tg)**	7.12	71	2.65%	0.0736	S1c
12.99	47 *	LRL31	9.46	59	-27.17%	**LRL146** (Line 1)	9.71	77	2.64%	0.3818	
10.79	93	LRL31	9.32	93	-13.62%	**LRL146** (Line 1)	9.29	89	-0.32%	0.4978	1b
9.48	74	LRL31	7.7	88	-18.78%	**LRL146** (Line 1)	7.91	86	2.73%	0.2631	
12.99	47 *	LRL31	9.46	59	-27.17%	**LRL147** (Line 2)	10.36	62	9.51%	< 0.0001	
10.79	93	LRL31	9.32	93	-13.62%	**LRL147** (Line 2)	9.98	77	7.02%	< 0.0001	S1d
9.48	74	LRL31	7.7	88	-18.78%	**LRL147** (Line 2)	8.7	75	12.99%	< 0.0001	
12.99	47 *	LRL31	9.46	59	-27.17%	**LRL148** (Line 3)	9.49	48 *	0.32%	0.9957	
10.79	93	LRL31	9.32	93	-13.62%	**LRL148** (Line 3)	8.93	82	-4.18%	0.0424	S1e
9.48	74	LRL31	7.7	88	-18.78%	**LRL148** (Line 3)	7.56	75	-1.82%	0.5542	

Supplemental Table 1. Lifespan analyses of neuronal HLH-30/TFEB rescued animals in *hlh-30(tm1878)* background. Animals were raised at 20ºC and grown at 25ºC on OP50. Where indicated (*), low number of events observed (EO, <50) were due largely due to censoring of animals which were more susceptible to internal progeny hatching at 25ºC. Statistical analyses: Mantel-Cox log-rank; Mean LS: Mean Lifespan. Refer to Supplemental Table 4 for additional strain information.

Supplemental Table 2

daf-2(e1370)III		***daf-2(e1379)III; hlh-30(tm1978)IV***			**Neuronal HLH-30 rescue strains**					
**Mean LS**	**EO**	**Mean LS**	**EO**	**% Difference to *daf-2(e1370)III***	**Strain**	**Mean LS**	**EO**	**% Difference to *daf-2(e1370)III; hlh-30(tm1978)***	***p* Value**	**Figure #**
31.52	95	15.28	69	-51.52%	**LRL167** (Line 1)	18.8	70	23.04%	< 0.0001	
28.35	72	13.06	50	-53.93%	**LRL167** (Line 1)	22.07	70	68.98%	< 0.0001	1c
33.67	80	13.26	61	-60.62%	**LRL167** (Line 1)	20.91	73	57.69%	< 0.0001	
31.52	95	15.28	69	-51.52%	**LRL168** (Line 2)	19.35	84	26.64%	< 0.0001	
28.35	72	13.06	50	-53.93%	**LRL168** (Line 2)	19.71	66	50.92%	< 0.0001	S2a
33.67	80	13.26	61	-60.62%	**LRL168** (Line 2)	23.08	65	74.06%	< 0.0001	
31.52	95	15.28	69	-51.52%	**LRL169** (Line 3)	18.07	61	18.26%	0.0009	
28.35	72	13.06	50	-53.93%	**LRL169** (Line 3)	19.69	52	52.35%	< 0.0001	S2b
33.67	80	13.26	61	-60.62%	**LRL169** (Line 3)	19.16	39 *****	44.40%	< 0.0001	

Supplemental Table 2. Lifespan analyses of neuronal *hlh-30* rescued animals in *daf-2(e1370);hlh-30(tm1978)* background. Animals were raised at 20ºC and grown at 25ºC on OP50. Where indicated (*), low number of events observed (EO, <50) were due largely due to censoring of animals which were more susceptible to internal progeny hatching at 25ºC. Statistical analyses: Mantel-Cox log-rank; Mean LS: Mean Lifespan. Refer to Supplemental Table 4 for additional strain information.

Supplemental Table 3

	Control RNAi				*daf-16* RNAi				
**Strain**	**Mean LS**	**EO**	**% Difference to *daf-2(e1370)III***	***p* Value**	**Mean LS**	**EO**	**% Difference to *daf-2(e1370)III***	***p* Value**	**Figure #**
daf-2(e1370)III	22.96	67			21.87	93			
daf-2(e1379)III; hlh-30(tm1978)IV	12.47	36 *	-45.69	< 0.0001	14.58	90	-33.33	< 0.0001	
**Neuronal HLH-30 rescue (LRL167)**	18.18	43 *	-20.82	0.0133	14.66	83	-32.97	< 0.0001	
daf-2(e1370)III	31.24	82			20.16	74			1d
daf-2(e1379)III; hlh-30(tm1978)IV	15.05	37 *	-51.82	< 0.0001	15.02	61	-25.5	< 0.0001	
**Neuronal HLH-30 rescue (LRL167)**	25.19	43 *	-19.37	0.0019	14.5	46 *	-28.08	< 0.0001	
daf-2(e1370)III	27.27	61			15.90	61			
daf-2(e1379)III; hlh-30(tm1978)IV	13.09	20 *	-52	< 0.0001	12.86	60	-19.11	< 0.0001	
**Neuronal HLH-30 rescue (LRL167)**	18.54	40 *	-32.01	< 0.0001	12.92	57	-18.74	< 0.0001	

Supplemental Table 3. Lifespan analyses of neuronal *hlh-30* rescued animals in *daf-2(e1370);hlh-30(tm1978)* background on *daf-16* RNAi. Animals developed at 20ºC on OP50 were transferred on day 1 of adulthood onto bacteria expressing RNAi and grown at 25ºC. Where indicated (*), low number of events observed (EO, <50) were largely due to censoring of animals which were more susceptible to internal progeny hatching at 25ºC. Statistical analyses: Mantel-Cox log-rank; Mean LS: Mean Lifespan. Refer to Supplemental Table 4 for additional strain information.

Supplemental Table 4

**Published strains used in this study**		
**Strain Name**	**Genotype**	**Strain Origin**
**N2-CK**	Wildtype	Hansen Lab
**CF1041**	*daf-2 (e1370)III*	Hansen Lab
**FX1978**	*hlh-30(tm1978)IV*	National BioSource Project
**FX4056**	*W06A11.1(tm4056)II*	National BioSource Project
**CF1038**	*daf-16(mu86)I*	Caenorhabditis Genetics Center (CGC)
**SD1347**	*cIs4251 [(pSAK2) myo-3p::GFP::LacZ::NLS + (pSAK4) myo-3p::mitochondrial GFP + dpy-20(+)]I*	Caenorhabditis Genetics Center (CGC)
**RW1596**	*myo-3(st386)V; stEx30 (myo-3p::GFP::myo-3 + rol-6(su1006))*	Caenorhabditis Genetics Center (CGC)
**CB1091**	*unc-13(e1091)I*	Caenorhabditis Genetics Center (CGC)
**DA509**	*unc-31(e928)IV*	Caenorhabditis Genetics Center (CGC)
**New strains used in this study**		
**Strain Name**	**Genotype**	**Comments**
**LRL31**	*hlh-30(tm1978)IV*	FX1978, 4X backcrossed to N2-CK
**LRL46**	*daf-2(e1370)III; hlh-30(tm1978)IV*	LRL31 x CF1041
**LRL72**	*hlh-30(tm1978)IV; llcEx31 (pLP11/hlh-30p::hlh-30::GFP::unc-54 3’UTR + pLP24/unc-122p::RFP)*	Refer to Methods for strain generation
**LRL81**	*hlh-30(tm1978)IV; llcEx33(pLP27/rab-3p::hlh-30::GFP::3XFLAG::rab-3 3'UTR + pLP24/unc-122p::RFP)*	Line 1; Refer to Methods for strain generation
**LRL82**	*hlh-30(tm1978)IV; llcEx34(pLP27/rab-3p::hlh-30::GFP::3XFLAG::rab-3 3'UTR + pLP24/unc-122p::RFP)*	Line 2; Refer to Methods for strain generation
**LRL83**	*hlh-30(tm1978)IV; llcEx35(pLP27/rab-3p::hlh-30::GFP::3XFLAG::rab-3 3'UTR + pLP24/unc-122p::RFP)*	Line 3; Refer to Methods for strain generation
**LRL130**	*hlh-30(tm1978)IV; daf-16(mu86)I*	LRL31 x CF1038
**LRL136**	*llcIs2(pLP27/rab-3p::hlh-30::GFP::3XFLAG::rab-3 3'UTR + pLP24/unc-122p::RFP)*	Refer to Methods for strain generation; 10X backcrossed to N2-CK
**LRL137**	*llcIs3(pLP27/rab-3p::hlh-30::GFP::3XFLAG::rab-3 3’UTR + pLP24/unc-122p::RFP)*	Refer to Methods for strain generation; 10X backcrossed to N2-CK
**LRL138**	*llcIs4 (pLP27/rab-3p::hlh-30::GFP::3XFLAG::rab-3 3'UTR + pLP24/unc-122p::RFP)*	Refer to Methods for strain generation; 10X backcrossed to N2-CK
**LRL146**	*hlh-30 (tm1978)IV; llcIs2(pLP27/rab-3p::hlh-30::GFP::3XFLAG::rab-3 3'UTR + pLP24/unc-122p::RFP)*	LRL31 X LRL136 (Line 1)
**LRL147**	*hlh-30 (tm1978)IV; llcIs3(pLP27/rab-3p::hlh-30::GFP::3XFLAG::rab-3 3'UTR + pLP24/unc-122p::RFP)*	LRL31 X LRL137 (Line 2)
**LRL148**	*hlh-30 (tm1978)IV; llcIs4(pLP27/rab-3p::hlh-30::GFP::3XFLAG::rab-3 3'UTR + pLP24/unc-122p::RFP)*	LRL31 X LRL138 (Line 3)
**LRL167**	*daf-2(e1370); hlh-30 (tm1978)IV; llcIs2(pLP27/rab-3p::hlh-30::GFP::3XFLAG::rab-3 3'UTR + pLP24/unc-122p::RFP)*	LRL146 X LRL46 (Line 1)
**LRL168**	*daf-2(e1370); hlh-30 (tm1978)IV; llcIs3(pLP27/rab-3p::hlh-30::GFP::3XFLAG::rab-3 3'UTR + pLP24/unc-122p::RFP)*	LRL147 X LRL46 (Line 2)
**LRL169**	*daf-2(e1370); hlh-30 (tm1978)IV; llcIs4(pLP27/rab-3p::hlh-30::GFP::3XFLAG::rab-3 3'UTR + pLP24/unc-122p::RFP)*	LRL148 X LRL46 (Line 3)
**LRL196**	*hlh-30(tm1978)IV; daf-16(mu86)I; llcIs3(pLP27/rab-3p::hlh-30::GFP::3XFLAG::rab-3 3'UTR + pLP24/unc-122p::RFP)*	LRL147 x LRL130
**LRL200**	*cIs4251 [(pSAK2) myo-3p::GFP::LacZ::NLS + (pSAK4) myo-3p::mitochondrial GFP + dpy-20(+)]I*	SD1347, 4X backcrossed to N2-CK

Supplemental Table 4. Strains used in the study

Supplemental Table 4 (continued)

New strains used in this study		
**Strain Name**	**Genotype**	**Comments**
**LRL201**	*hlh-30 (tm1978)IV; cIs4251 [(pSAK2) myo-3p::GFP::LacZ::NLS + (pSAK4) myo-3p::mitochondrial GFP + dpy-20(+)]I*	LRL31 x LRL200
**LRL204**	*myo-3(st386)V; stEx30 (myo-3p::GFP::myo-3 + rol-6(su1006))*	RW1596, 4X backcrossed to N2-CK
**LRL207**	*W06A11.1(tm4056)II*	FX4056, 4X backcrossed to N2-CK
**LRL208**	*hlh-30(tm1978)IV; myo-3(st386)V; stEx30 (myo-3p::GFP::myo-3 + rol-6(su1006))*	LRL31 x LRL204
**LRL212**	*hlh-30 (tm1978)IV; llcIs3(pLP27/rab-3p::hlh-30::GFP::3XFLAG::rab-3 3'UTR + pLP24/unc-122p::RFP); cIs4251 [(pSAK2) myo-3p::GFP::LacZ::NLS + (pSAK4) myo-3p::mitochondrial GFP + dpy-20(+)]I*	LRL147 x LRL201
**LRL216**	*hlh-30(tm1978)IV; W06A11.1(tm4056)II*	LRL31 x LRL207
**LRL217**	*hlh-30 (tm1978)IV; llcIs3(pLP27/rab-3p::hlh-30::GFP::3XFLAG::rab-3 3'UTR + pLP24/unc-122p::RFP); myo-3(st386)V; stEx30 (myo-3p::GFP::myo-3 + rol-6(su1006))*	LRL147 x LRL208
**LRL218**	*hlh-30(tm1978)IV; W06A11.1(tm4056)II*; *llcIs3(pLP27/rab-3p::hlh-30::GFP::3XFLAG::rab-3 3'UTR + pLP24/unc-122p::RFP)*	LRL147 x LRL216
**LRL219**	*unc-13(e1091)I*	CB1091, 4X backcrossed to N2-CK
**LRL220**	*unc-31(e928)IV*	DA509, 4X backcrossed to N2-CK
**LRL222**	*hlh-30(tm1978)IV; unc-13(e1091)I*	LRL31 x LRL219
**LRL223**	*unc-13(e1091)I; cIs4251 [(pSAK2) myo-3p::GFP::LacZ::NLS + (pSAK4) myo-3p::mitochondrial GFP + dpy-20(+)]I*	LRL200 x LRL219
**LRL227**	*hlh-30 (tm1978)IV; llcIs3(pLP27/rab-3p::hlh-30::GFP::3XFLAG::rab-3 3'UTR + pLP24/unc-122p::RFP); unc-13(e1091)I*	LRL147 x LRL222
**LRL228**	*unc-31(e928)IV; cIs4251 [(pSAK2) myo-3p::GFP::LacZ::NLS + (pSAK4) myo-3p::mitochondrial GFP + dpy-20(+)]I*	LRL200 x LRL220
**LRL229**	*hlh-30(tm1978)IV; unc-31(e928)IV*	LRL31 x LRL220
**LRL230**	*hlh-30 (tm1978)IV; llcIs3(pLP27/rab-3p::hlh-30::GFP::3XFLAG::rab-3 3'UTR + pLP24/unc-122p::RFP); unc-31(e928)IV*	LRL147 x LRL229
**LRL231**	*hlh-30 (tm1978)IV; cIs4251 [(pSAK2) myo-3p::GFP::LacZ::NLS + (pSAK4) myo-3p::mitochondrial GFP + dpy-20(+)]I; W06A11.1(tm4056)II*	LRL201 x LRL216
**LRL232**	*hlh-30 (tm1978)IV; llcIs3(pLP27/rab-3p::hlh-30::GFP::3XFLAG::rab-3 3'UTR + pLP24/unc-122p::RFP); cIs4251 [(pSAK2) myo-3p::GFP::LacZ::NLS + (pSAK4) myo-3p::mitochondrial GFP + dpy-20(+)]I; W06A11.1(tm4056)II*	LRL212 x LRL218
**LRL238**	*hlh-30 (tm1978)IV; llcIs3(pLP27/rab-3p::hlh-30::GFP::3XFLAG::rab-3 3'UTR + pLP24/unc-122p::RFP); cIs4251 [(pSAK2) myo-3p::GFP::LacZ::NLS + (pSAK4) myo-3p::mitochondrial GFP + dpy-20(+)]I; unc-31(e928)IV*	LRL212 x LRL230
**LRL239**	*W06A11.1(tm4056)II; cIs4251 [(pSAK2) myo-3p::GFP::LacZ::NLS + (pSAK4) myo-3p::mitochondrial GFP + dpy-20(+)]I*	LRL200 x LRL207
**LRL240**	*hlh-30 (tm1978)IV; llcIs3(pLP27/rab-3p::hlh-30::GFP::3XFLAG::rab-3 3'UTR + pLP24/unc-122p::RFP); cIs4251 [(pSAK2) myo-3p::GFP::LacZ::NLS + (pSAK4) myo-3p::mitochondrial GFP + dpy-20(+)]I; unc-13(e1091)I*	LRL212 x LRL227
**LRL241**	*hlh-30 (tm1978)IV; cIs4251 [(pSAK2) myo-3p::GFP::LacZ::NLS + (pSAK4) myo-3p::mitochondrial GFP + dpy-20(+)]I; unc-31(e928)IV*	LRL201 x LRL229
**LRL242**	*hlh-30 (tm1978)IV; cIs4251 [(pSAK2) myo-3p::GFP::LacZ::NLS + (pSAK4) myo-3p::mitochondrial GFP + dpy-20(+)]I; unc-13(e1091)I*	LRL201 x LRL222

Supplemental Table 4. Strains used in the study

Supplemental Table 4 (continued)

New strains used in this study		
**Strain Name**	**Genotype**	**Comments**
**LRL244**	*llcEx61 (pLP30/W06A11.1p::W06A11.1::DsRed + pLP7/myo-2p::GFP)*	Line 1; Refer to Methods for strain generation
**LRL245**	*llcEx62 (pLP30/W06A11.1p::W06A11.1::DsRed + pLP7/myo-2p::GFP)*	Line 2; Refer to Methods for strain generation
**LRL247**	*W06A11.1(tm4056)II; unc-13(e1091)I; cIs4251 [(pSAK2) myo-3p::GFP::LacZ::NLS + (pSAK4) myo-3p::mitochondrial GFP + dpy-20(+)]I*	LRL207 x LRL223
**LRL248**	*llcEx61 (pLP30/W06A11.1p::W06A11.1::DsRed + pLP7/myo-2p::GFP); cIs4251 [(pSAK2) myo-3p::GFP::LacZ::NLS + (pSAK4) myo-3p::mitochondrial GFP + dpy-20(+)]I*	LRL200 x LRL244
**LRL253**	*W06A11.1(tm4056)II; llcEx61 (pLP30/W06A11.1p::W06A11.1::DsRed + pLP7/myo-2p::GFP)*	LRL207 x LRL244

Supplemental Table 4. Strains used in the study

Supplemental Table 5

Primers used for cloning			
**Primer**	**Direction**	**Sequence (5' to 3')**	**Purpose**
LC127	Forward	GGTGGTGGTACCATGGCCGGCGATTATAAGGA	Cloning of 3xFLAG-tag from pLP15 (Addgene plasmid #55180) with 5' KpnI and 3' EcoRI sites, for insertion into KpnI/EcoRI-digested pLP9 (Addgene plasmid #1497).
LC128	Reverse	GGTGGTGAATTCTTAACCGGTCTTGTCGTCATCG	
LC154	Forward	TCAGGAGGACCCTTGGAGGGTACGGTACCATGGCCGATGAC	Cloning of *hlh-30* coding sequence from *C. elegans* cDNA with 5' KpnI and 3' NaeI sites, for insertion into KpnI/NaeI-digested pLP16 (pPD95.81_*3XFLAG)* vector.
LC155	Reverse	TCATGATCCTTATAATCGCCCGAAAAGTCCATGTGATAATGACC	
LC184	Forward	GCCCGAAAAGTCCATGTGATAATG	Cloning of pPD95.81_*hlh-30::3xFLAG*, for insertion of LC186/187-amplified *GFP* sequence between *hlh-30* and *3xFLAG* sequences via HiFi cloning.
LC185	Reverse	GATTATAAGGATCATGATGGTG	
LC186	Forward	CATTATCACATGGACTTTTCGGGCTCGGGCTCGATGAGTAAAGG AGAAGAAC	Cloning of *GFP* sequence from pLP19 (plasmid #836 (*hlh-17p::GFP*) from Dr. Andrew Dillin, UC Berkeley), for insertion between *hlh-30* and *3xFLAG* sequences of LC184/185-amplified pPD95.81_*hlh-30::3xFLAG* vector via HiFi cloning.
LC187	Reverse	CACCATCATGATCCTTATAATCCGAGCCCGAGCCTTTGTATAGTT CATCCATGCCATG	
LC143	Forward	GGTGGTGCATGCGATCTTCAGATGGGAGCAGTG	Cloning of *rab-3* promoter from pLP17 (plasmid #462 (*rab-3p::sid-1*) from Dr. Andrew Dillin, UC Berkeley) with 5' SphI and 3' KpnI sites, for insertion into SphI/KpnI-digested pPD95.81_*hlh-30::GFP::3xFLAG* vector.
LC188	Reverse	GGTGGTGGTACCCTGAAAATAGGGCTACTGTAG	
LC225	Forward	GCGGCCCCTATTATTTTTGACACC	Cloning of pLP21 (pPD95.81_*rab-3p::hlh-30::3xFLAG*) vector, for replacement of *unc-54* 3'UTR sequence with LC227/228-amplified *rab-3 3'UTR* sequence via HiFi cloning.
LC226	Reverse	CACAAGTATTGATGAGCACGATGC	
LC227	Forward	GGTGTCAAAAATAATAGGGGCCGCGAAGCTCGAAGCGAATCC	Cloning of *rab-3 3'UTR* from *C. elegans* gDNA, for replacement of *unc-54* 3'UTR sequence in LC225/226-amplified pPD95.81_*rab-3p::hlh-30::3xFLAG* vector via HiFi cloning.
LC228	Reverse	GCATCGTGCTCATCAATACTTGTGCAGACCTCTGGAACTCTTC	
LC341	Forward	ATGGTGCGCTCCTCCAAGAAC	Cloning of pLP26 (*p62p::p62::dsRED*) vector for the replacement of *p62p::p62* sequences with LC345/346-amplified *W06A11.1* coding and LC353/354-amplified *W06A11.1* promoter sequences via HiFi cloning.
LC342	Reverse	GTTAGCGTATCCATCGTTGTGAGTG	
LC345	Forward	ATGATCCGGCCATTACCATTTCTTC	Cloning of *W06A11.1* coding sequence from *C. elegans* cDNA for the replacement of *p62p::p62* sequences in LC341/342-amplified pLP26 (*p62p::p62::dsRED*) vector via HiFi cloning.
LC346	Reverse	GTTCTTGGAGGAGCGCACCATCGAGCCCGAGCCGAGCAAACGA AGAATTGAGATGAC	
LC353	Forward	CACTCACAACGATGGATACGCTAACGAGAGCGGAAGACGATTT TGGAGATAGACAGTG	Cloning of *W06A11.1* promoter sequence from *C. elegans* gDNA for the replacement of *p62p::p62* sequences in LC341/342-amplified pLP26 (*p62p::p62::dsRED*) vector via HiFi cloning.
LC354	Reverse	GAAATGGTAATGGCCGGATCATAATGTGTGCTCTGTGATGTAA CTGGC	

Supplemental Table 5. Primers used in this study

Supplemental Table 5 (continued)

Primer	Direction	Sequence (5' to 3')	Purpose
**Primers used for genotyping**			
LC106	Forward	CAGATCCTCCTCCTACTTTCC	Genotyping of *hlh-30(tm1978)* deletion. WT = 1273 bp, *hlh-30(tm1978)* = 563 bp
LC80	Reverse	CTAGCCGATCCGACCGAGAA	
LC203	Forward	GCGTACTCCTCATCCAGCGATC	Amplification of gDNA region with *daf-2(e1370)* point mutation (726 bp)
LC204	Reverse	CCATCGAGATCTCGCCGC	
LC199	Forward	GAATCCGTATTCCGACGTTC	Confirmation of *daf-2(e1370)* mutation in LC203/204 amplicons by sequencing
LC231	Forward	CACTGTCTACCTCTCCTCCTG	Genotyping of *daf-16(mu86)* deletion. LC231/232 = WT (693 bp), LC231/233 = *daf-16(mu86)*; based on thermocycling conditions used in this study
LC232	Reverse	GCGTCAGTTCCGATCTGATATGAAC	
LC233	Reverse	CGTTATCAAATGCTCCTTGCATTGAATC	
LC294	Forward	GCCCAACATCTTCCCAAGCATG	Amplification of gDNA region with *unc-13(e1091)* point mutation (328 bp) and allele confirmation by sequencing with LC294
LC295	Reverse	CTCTTCCTGTCCTTCTTCGTAGCC	
LC296	Forward	AACCAAGACGACCGATCAGT	Genotyping of *W06A11.1(tm4056)* deletion. WT = 780 bp, *W06A11.1(tm4056)* = 494 bp
LC297	Reverse	GGCGCACGAACACACGCATA	
LC312	Forward	GCCACAGGTCAAGCCTATAA	Genotyping of *unc-31(e928)* deletion. WT = 1197 bp, *unc-31(e928)* = no product
LC313	Reverse	TGAGCCGGACTAACATCAATAC	
**Primers used for qPCR**			
**Primer**	**Direction**	**Sequence (5' to 3')**	**Purpose**
LL7	Forward	TGGAACTCTGGAGTCACACC	For comparing expression levels of the housekeeping gene, *ama-1*. Used for normalization.
LL8	Reverse	CATCCTCCTTCATTGAACGG	
LL9	Forward	CTGCTGGACAGGAAGATTACG	For comparing expression levels of the housekeeping gene, *cdc-42*. Used for normalization.
LL10	Reverse	CTCGGACATTCTCGAATGAAG	
LL11	Forward	GTTCCCGTGTTCATCACTCAT	For comparing expression levels of the housekeeping gene, *pmp-3*. Used for normalization.
LL12	Reverse	ACACCGTCGAGAAGCTGTAGA	
LL624	Forward	CCGACGAGTTCGATCGAC	For comparing *hlh-30* expression levels
LL626	Reverse	GTCGGCGTTCAATCATATTGTG	

Supplemental Table 5. Primers used in this study
